# CD44-Targeted Nanocarrier for Cancer Therapy

**DOI:** 10.3389/fphar.2021.800481

**Published:** 2022-03-31

**Authors:** Prashant Kesharwani, Rahul Chadar, Afsana Sheikh, Waleed Y. Rizg, Awaji Y Safhi

**Affiliations:** ^1^ Department of Pharmaceutics, School of Pharmaceutical Education and Research, Jamia Hamdard, New Delhi, India; ^2^ Department of Pharmaceutics, Faculty of Pharmacy, King Abdulaziz University, Jeddah, Saudi Arabia; ^3^ Department of Pharmaceutics, Faculty of Pharmacy, Jazan University, Jazan, Saudi Arabia

**Keywords:** CD44, targeted drug delivery, gene delivery, hyaluronic acid, anticancer therapy, nanocarrier

## Abstract

Cluster of differentiation 44 (CD44) is a cell surface glycoprotein overexpressed in varieties of solid tumors including pancreatic, breast, ovary, brain, and lung cancers. It is a multi-structural glycoprotein of the cell surface which is majorly involved in cell proliferation, cell-to-cell interaction, cellular migration, inflammation, and generation of immune responses. Numerous studies focus on the development of nanocarriers for active targeting of the CD44 receptor to improve efficacy of targeting chemotherapy and achieve precise chemotherapy by defining the release, uptake, and accumulation of therapeutic agents. The CD44 receptor has a selective binding affinity towards hyaluronic and chondroitin sulfate (CS). Taking this into consideration, this review focused on the role of CD44 in cancer and its therapy using several nanocarriers such as polymeric/non-polymeric nanoparticles, dendrimer, micelles, carbon nanotubes, nanogels, nanoemulsions etc., for targeted delivery of several chemotherapeutic molecules and nucleic acid. This review also illuminates the role of hyaluronic acid (HA) in cancer therapy, interaction of HA with CD44, and various approaches to target CD44-overexpressed neoplastic cells.

## Introduction

Among the recent statistics of most life-threatening diseases, cancer poses a major issue by being the most dangerous and fatal disease causing the death of millions of people worldwide every year; the number of deaths due to cancer is gradually rising. Cancer is the major challenge of the 21st century, that does not have any limit, it can occur in any organ of the body and can migrate to nearby tissue (Bharali and Mousa, 2010). Cancer can originate in any part of the human body, comprising of trillions of cells. In general, cells grow, multiply, become old/damaged, die, and new cells replace the old cells, but in the case of cancer, this orderly process breaks down, causing tumors or lumps of tissue (Kateb et al., 2011; Singh S. et al., 2021). The process of cancer treatment is very complex because of its molecular complexity at genetic and phenotypic levels. Due to molecular complexity, cancer cells exhibit clinical diversity and therapeutic resistance. There are numerous strategies, such as surgical removal, radiation therapy, chemotherapy, and hormonal therapy, that have been developed for cancer treatment but each of them possesses drawbacks and side effects. Chemotherapy is the most commonly used anticancer therapy for cancer management (Kamal et al., 2012; Zhao and Rodriguez, 2012; Sheikh and Kesharwani, 2021). In most cases, due to non-specific delivery, chemotherapeutic agents fail to deliver desired therapeutic effects along with generating multiple side effects (Chadar et al., 2021; Kaur and Kesharwani, 2021; Singh and Kesharwani, 2021). Development of cancer therapy is a very complex, challenging, and expensive process, thus, the future of cancer therapy is associated with cutting edge research in polymer chemistry and electronic engineering. Differentiation between cancerous and normal cells poses another challenge for physicians and oncology scientists. Therefore, engineering of drugs in such a framework can recognize tumor cells to inhibit cell growth and proliferation. Conventional chemotherapy (CC) interacts with normal tissues causing numerous undesired effects including organ failure/damage (Mousa and Bharali, 2011).

To overcome the challenges associated with conventional chemotherapy, currently various evolving nanotechnology-based nanocarriers have been developed. Several nanocarriers such as dendrimers, liposomes, micelles, nanogels, emulsions, carbon nanotubes, polymeric/non-polymeric nanoparticles, and quantum dots displayed great potential to deliver chemotherapeutic agents at the target of interest. The potential of nanocarriers includes improvement in physicochemical properties of chemotherapeutic molecules, reduced side effects, targetability, reduced doses of drugs, enhanced blood circulation time, and many others. Nanoparticles (NPs) can be used for both passive targeting and active targeting, but passive targeting is associated with some limitations, so that development of actively targeting NPs or “intelligent” NPs is of utmost importance as they can deliver their cargo to cancerous cells by binding with overexpressed biomarkers present on the surface of cancer cells. As mentioned earlier, the molecular structure of cancer is different from the normal cell. Several receptors such as integrin, folate receptors (FR), transferrin, EGFR, sigma, GPCR, and CD44 are overexpressed in tumor cells. Thus, development of a system to target such cells could provide a way for efficient and active delivery of chemotherapeutic agents.

**FIGURE 1:**
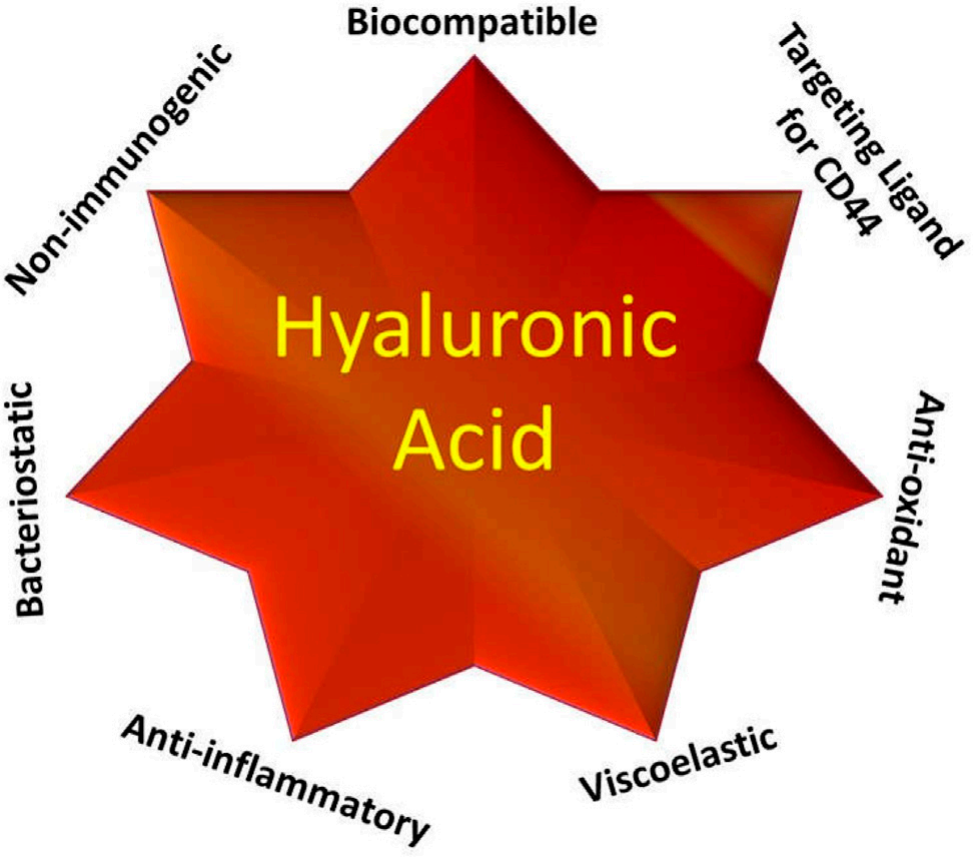
Potential of hyaluronic acid in pharmaceutical drug delivery.

Cluster of differentiation 44 (CD44) is overexpressed in several types of cancer including breast, lungs, ovary, brain, and hepatic carcinoma which are responsible for many metabolic activities of cancer cells such as cellular differentiation, migration, proliferation hematopoiesis, angiogenesis, and cell and tumor metastasis (Kesharwani et al., 2015a; Kesharwani et al., 2015b). The CD44 receptor after binding with hyaluronic acid (HA), is activated and potentiates cancer cell growth. Utilizing such a process of tumor cell growth, researchers concluded that the CD44 receptor could be a promising target for anticancer therapy (Goodison et al., 1999; Luo et al., 2019). The CD44 receptor has a remarkable affinity towards glycosaminoglycan polymers like HA and chondroitin sulfate (CS). HA, a biocompatible, biodegradable, low immunogenic hydrophilic polymer is mainly located in the epithelial, neural, and connective tissue (Salari et al., 2021) (Figure 1). Most importantly, HA could bind to the CD44 receptor *via* H-bond or van der Waals forces, depending on the variant and expression level of the CD44 receptor (Banerji et al., 2007; Dosio et al., 2016; Kim et al., 2019; Spadea et al., 2019; Li et al., 2021). The HA molecule contains several functional groups such as -COOH, -OH and N-acetyl groups through which it could easily be conjugated with different types of nanomaterials and with different chemotherapeutics drugs like doxorubicin (DOX), paclitaxel (PTX), docetaxel (DTX), and camptothecin (CPT) (Cai et al., 2019; Luo et al., 2019). CS is another ligand for targeting the CD44 receptor with a similar chemical structure to HA, thereby drawing interest of formulation scientists towards such targeting ligands. Likewise, CS also possesses many functional groups through which it can conjugate with different nanomaterials and drugs. In a study, Onishi et al. reported that DOX and PTX conjugate with CS particles (Lee et al., 2016a; Jin et al., 2017; Onishi et al., 2019). It is important to understand the binding ability of HA with its receptor. Low molecular weight HA binds to monomeric CD44 receptors and activates TLR 2/4 receptors showing a pro-inflammatory characteristic, while the high molecular weight HA inhibits TLR activation and induces an anti-inflammatory characteristic (Lesley et al., 2000). Moreover, the receptor binding of HA depends on the following factors: HA is required to bind at multiple sites as a single interaction could be weak, for binding with more than one receptor, more than 20 residues of oligosaccharide are required and the molecular weight of HA should be more than 31 kDa as one below 31 kDa can attach with a single receptor while those above 132 kDa could interact with five to eight receptors (Oommen et al., 2014).

### Structure of the Target CD44

The type I transmembrane receptor protein known as CD44 bears a common structure consisting of four major domains: the stem region, the cytoplasmic region, extracellular domain, and the transmembrane region (Dzwonek and Wilczyński, 2015). The cytoplasmic domain of CD44 constitutes both a short and long tail that demonstrate its ability in the transcription process and nuclear localization (Naor et al., 2008). The extracellular domain interacts and senses the stimuli with/in the external microenvironment (Hill et al., 2006). The transmembrane domain provides a platform for interaction of the adaptor protein with co-factors. It also helps in directing the homing of lymphocytes (Williams et al., 2013).

The target protein of CD44 is encoded by single genes comprising of 20 exons. The endothelial cells, fibroblast, neurons, and leukocytes along with many vertebrate cells show high expression of exons 1–5, 16–18, and 20 (Naor et al., 2008). The exons between 6–15 present in the middle of the CD44 gene are spliced alternatively to form a variant of CD44 (CD44V) to develop its isoform showing variable function in the stem region (Naor et al., 1997). The amino group present on the periphery of exons basically contains two binding sites; the linking site comprising of 32–132 amino acids and exterior binding motif to the linking site that comprises 150–158 amino acid groups. In between the N-terminal globular domain and the transmembrane site, there lies 46 amino acids in a stretch forming a star-shaped structure. This shape contains numerous cleavable sites which are extra-glycosylated. The stretch could further be modified by inserting various exons (Kalniņa et al., 2005). The cytoplasmic tail and *O*-glycosylation demonstrates its localization on the cell membrane and thus enables the crosstalk between HA and CD44 (Neame and Isacke, 1993). The CD44 isoforms can be modified through fabrication with O-glycans, N-glycans, and glycosaminoglycans, such as chondroitin sulfate and heparan sulfate (Greenfield et al., 1999; Gomari et al., 2021). The binding of CD44 with its agonist and antagonist is dependent on the activation state of the CD44 receptor. Apart from binding with HA, CD44 also interact with the extracellular proteins such as matrix metalloproteinases (MMPs), growth factors, fibronectin, cytokines, collagens, and chemokines (Ma et al., 2019).

## Nanotechnology in Cancer Targeting

Nanotechnology is an umbrella term defined as the scientific principles, process, methodologies, techniques, and other scientific activity dealing in the size range from a few nanometers to several hundred nm, depending upon their intended use (Peer et al., 2007; Dubey et al., 2021; Srivastava et al., 2021; Kumar et al., 2021). Nanotechnology has become the focused area of interest over the last few decades for emerging drug delivery systems because it offers several unique advantages over the conventional drug delivery system (Malam et al., 2009; Zeeshan et al., 2021). It is also a promising and intelligent approach for the theranosis of many dangerous diseases like cancer, as it is able to deliver its cargo directly to its target. Nanotechnology is actively implemented in several cancer therapies mainly for detection, diagnosis, imaging, and treatment of different types of cancers. A significant amount of research and advancement is being carried out to find the most precise cancer treatment with minimal side effects encountered by conventional therapy. Multiple nanocarriers have been designed to modify therapeutic drugs in such a way that could overcome limitations such as biological barrier, unintended intermolecular interactions, and distribution to many intact or normal tissues. Nanoparticles’ unique characteristics such as being modifiable could be utilized for optical, magnetic, electronic, and biological observation offering several advantages over macro nanoparticles (Park, 2007; Praetorius and Mandal, 2008; Patnaik et al., 2021; Surekha et al., 2021).

**FIGURE 2:**
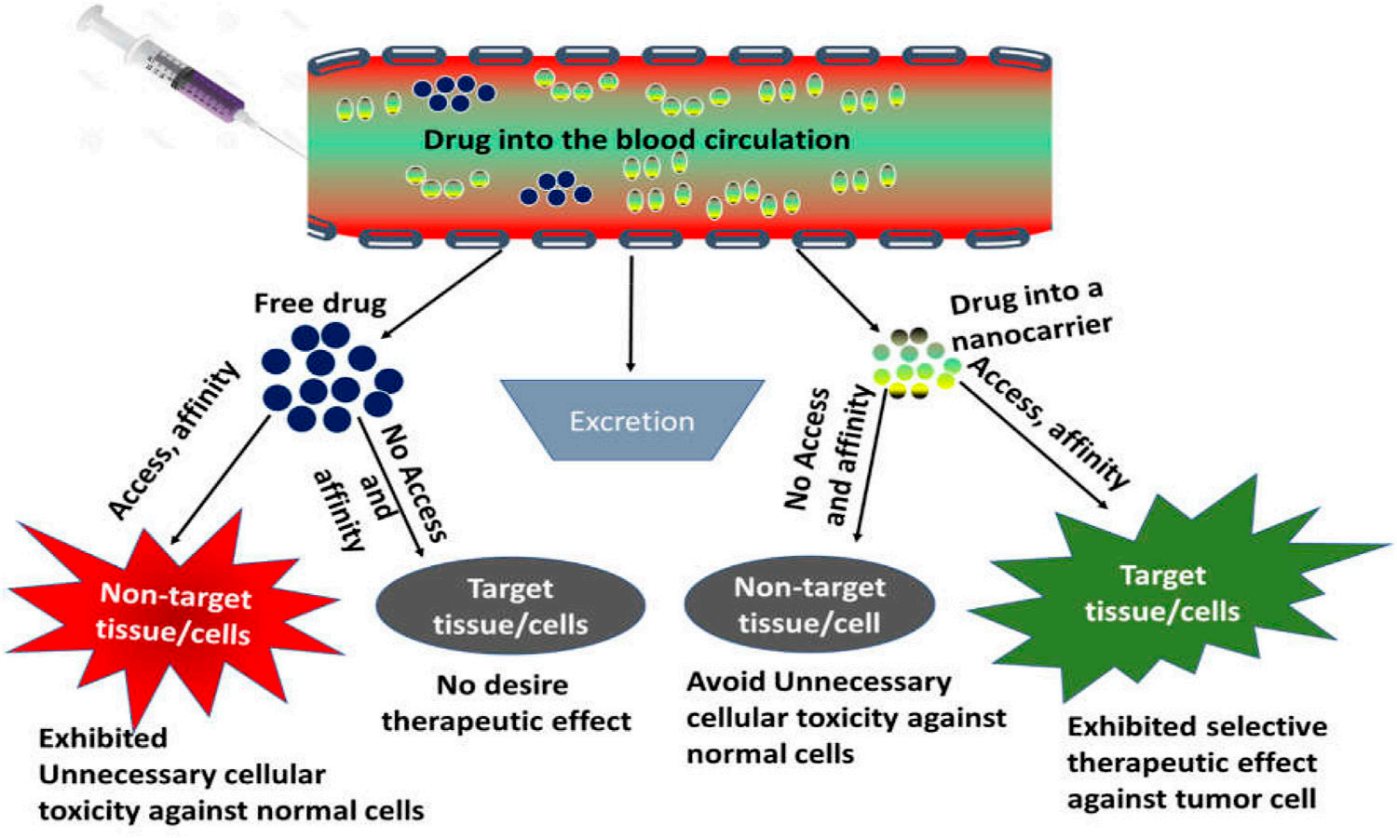
Basic principle of drug targeting.

Nanotechnology has revolutionized the field of biomedicine, especially in cancer therapy for selective targeting of tumor cells. Characteristics of several nanocarriers can be modified such as size, shape, and texture by chemical or physical modification, making them important candidates for desired delivery action at specific sites. Nanoparticles could be programmed towards selective tumor cells either *via* active targeting or passive targeting (Singh V. et al., 2021; Gupta et al., 2021; Zafar et al., 2021; Zhang et al., 2021) (Figure 2).

### Nanotechnology-Based Active Drug Targeting

Active targeting is the delivery of drugs and other therapeutic molecules directly to the tumor cells. For active targeting, chemotherapeutic-loaded nanocarriers have been framed in a such way that they can directly interact with or recognize tumor cells. Several pathophysiological changes of cancers cells form the basis of active targeting by recognizing overexpressed biomarkers and genes. Basically, nanoparticle surfaces can be decorated with ligands which can easily be recognized by tumor cells, that act either by ligand receptor binding or antigen-antibody binding (Guo and Szoka, 2003; Cho et al., 2008; Jin et al., 2020). A targeting drug delivery system based on nanotechnology mainly consist of three parts, first, cell death-causing agents (chemotherapeutic drugs), second, targeting ligand/penetration enhancers, and third, a nanocarrier. Today, numerous materials are used to construct nanocarriers but most commonly used materials include polymers (Kavand et al., 2020), lipids (Shankar et al., 2018), ceramics (Cisterna et al., 2016), and metals (Yezhelyev et al., 2006; Cho et al., 2008; Hitchcock et al., 2020). Usually, natural and synthetic polymers and lipids are most commonly used as vectors for drug delivery (Duncan, 2006; Schroeder et al., 2012). Early clearance of nanoparticles from circulation *via* an RES system is a major limitation of nanocarriers, which can be solved by polymeric modification. NPs modified by hydrophilic polymers allows them to remain in the circulation system for a long period of time or for the duration of NPs’ interaction with cancerous cells. Due to surface modification, hydrophilic nanoparticles avoid cellular opsonization by repelling plasma proteins (Jeon et al., 1991; Brigger et al., 2002; Francis et al., 2004). There are innumerable hydrophilic polymers available that are used for the targeted delivery of chemotherapeutics, a few examples of such polymers include poly ethylene glycol (PEG), poloxamines, poloxamers, polysaccharides (hyaluronic acid), etc. (Storm et al., 1995; Torchilin and Trubetskoy, 1995). As mentioned earlier, the molecular structure of cancer cells is different from healthy cells, that show overexpression of some receptors on their surface making them selectively targetable for drug delivery. When a ligand-modified nanocarrier is administered, it is easily recognized by the overexpressed receptor on tumor cells that can be identified, bound, and rapidly internalized by receptor-mediated endocytosis or phagocytosis of tumor cells. Once well-decorated NPs are internalized into the tumor cell, the NPs release their cargo intracellularly, which leads to apoptosis (Peer et al., 2007). Receptors such as the folate receptor, CD44 receptor, transferrin receptor, luteinizing hormone-releasing hormone receptor, and asialo glycol protein (ASGP), are some examples of receptors which are most often discussed for cancer targeting therapy.

### Nanotechnology-Based Passive Targeting

As reported in several studies, the metabolic activities of tumor cells rise to fulfil the energy demand required by rapidly growing tumor cells. The blood vessels linked with tumor cells form leaky vasculature due to basement membrane irregularity, which enhances the penetration of molecules to pass through the blood vessel. The size of this leaky vasculature ranges from 100–780 nm, so the NPs below this size can easily pass across the leaky vasculature of tumor cells. Due to lack of a well-defined lymphatic network, the retention time of the entered drug into the tumor interstitium increases, facilitating the EPR effect, that could enhance drug accumulation. It is well reported that NPs deliver their therapeutic effect *via* both active and passive targeting approaches (Maeda et al., 2000; Maeda, 2001; Alavi and Hamidi, 2019).

## Role of CD44 in Cancer Targeting

**FIGURE 3:**
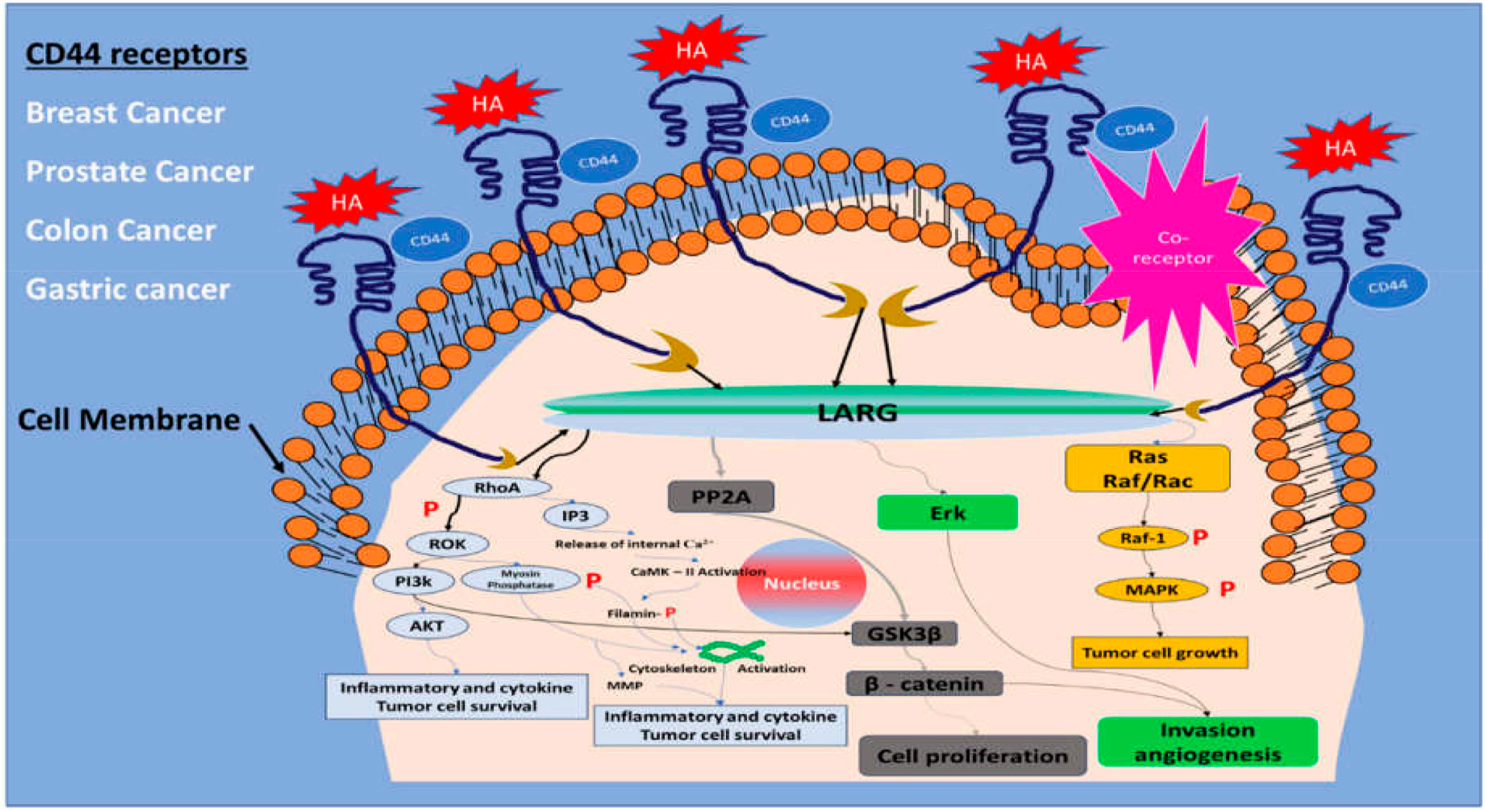
Cell signaling pathways associated with the CD44 receptor.

CD44 is a kind of glycoprotein overly expressed in a variety of mammalian tumor cells including prostate, breast, colon, gastric, head, and squamous cell carcinoma. It is also majorly involved in a number of non-cancerous diseases such as arthritis, bacterial and viral infections, lung disease, wound healing, and cardiovascular issues (Sherman et al., 1994). CD44 cells are associated with a wide variety of vital cellular functions like lymphocyte activation, recirculation, hematopoiesis, cell division, migration, adhesion, and signaling, etc. CD44 cells are comprised of nine variable axons 1–5 and 16–20, resulting in the formation of a variety of CD44 splice variants (Cao H. et al., 2016). Inflammatory response and cellular damage alter the cellular expression and functions of the CD44 cells. Oxley and Sackstein established a relation between CD44 cell antigens with cell-cell interactions, downstream signaling, cell adhesion, and proliferation (Oxley and Sackstein, 1994). The non-sulfated glycosaminoglycan hyaluronic acid (HA) is the most common or principle substrate of CD44 cells; other than HA, osteopontin, collagens, and matrix metalloproteinase (MMP) also interact with CD44 receptors (Senbanjo and Chellaiah, 2017). Such a substrate plays an important role in the regulation of cell signaling in accordance with several other factors such as post-translational modification, varied expression of isoform, along with spatial distribution of the CD44 receptor on the cell surface that contributes to control CD44 functions (Misra et al., 2015). The thrust areas that determine the role of CD44 in cancer metastasis and growth are divided into two main categories, one is HA-dependent, and another is HA-independent. HA-independent cancer signaling of CD44 cells depends on the interactions of the intracellular domain of CD44 cells and cytoskeletal proteins or kinases. HA-dependent signaling relies on the interaction of its native ligand HA with CD44 cells, almost all isoform of CD44 cells show affinity for its most specific and robust ligand, HA. The stem cell niches mainly comprise of HA, which is a chief constituent of the cancer matrix. CD44 is strongly associated with matrix assembly, thus the association of CD44 and HA could not only aid in arresting cancerous cells but also facilitate modification of tissue matrix to maintain colonization (Hill et al., 2006; Kuhn and Tuan, 2010). Matrix component perturbation could alter the cell shape and intracellular tension, exhibiting shifting of signaling events that modify gene expression (Wang et al., 2009). CD44 isoforms such as CD44v3, CD44v5, and CD44v6 are also involved in matrix assembly whereas CD44v5, CD44v6, and CD44v7-8 play key roles in lymph node metastasis (Jung et al., 2009). The isoform of CD44 encodes for various peptides inside the juxta membrane domain causing conformational changes along with providing binding sites for growth factors and cytokines. Such binding mediates tumor growth and associated activities such as prognosis and metastasis. Many studies identified tumor cells with stem-like characteristics [cancer stem cells (CSCs)] that have self-renewal, tumor metastatic, and recurrent characteristics. In many cancers such as colon and breast cancer, CD44 acts as a significant marker on CSCs, however in many situations the specific isoforms are still unknown (Zeilstra et al., 2008). CD44v6 isoforms have been investigated as colon cancer stem cell markers with metastatic propensity suggesting CD44v6 targeting in colon tumors could potentially provide significant results. However, CD44 isoforms are also present in tumor cells that respond to drugs as well. However, to improve the tumor homing effect, a ligand is required (Alves and Konstantopoulos, 2012). Several metabolic pathways like PI3K/Akt, PPA2, Erk, and Ras/Raf are also associated with CD44 cell signaling (Figure 3). Such pathways regulate inflammation/cytokines through the NF-ĸB pathway, proliferation through B-catenin, invasion/angiogenesis, and cytoskeleton rearrangement, respectively (Chen et al., 2018). Isoforms CD44/CD44-V6 are also reported as important anti-apoptotic genes. Expression of CD44-V9 and CD44-V6 are mainly controlled by cytokines like TNFα and IFN3. CD44 plays key roles in tumor development through the above metabolic pathways and other physiological events. Under normal conditions, CD44 regulates normal function and maintains cellular homeostasis. But in the case of a tumor, it fails to maintain such cellular function. As described earlier, CD44 cells have high affinity for several ligands, HA is considered as one of the best ligands for CD44. Interaction of HA with CD44 activates the FAK-linked PI3K/Akt and ERK signaling pathways (Misra et al., 2011). Various studies reported a high chance of CD44 isoform transition in tumor cells, thereby developing different types of cancers by the activation of HA. Overexpression of CD44-V6 and CD44-V10 isoforms are also associated with colorectal carcinoma. The CD44-V6 isoform is the strongest and most active variant among all other variants that are involved in cell migration and metastasis activated by the Wnt/B-catenin pathway (Thapa and Wilson, 2016). The upregulated CD44-V6 shows high viability in comparison to its other variants after treatment with standard chemotherapy. In breast cancer, the role of CD44 depends upon many factors, like CD44 expression, ligand binding ability, cell variants, methylation/splicing events, etc. (Afify et al., 2009). Bourguignon et al. studied the interaction of HA with CD44 causing expression of Pgp (MDR) along with the anti-apoptotic Bcl gene in breast cancer, thereby mediating proliferation and survival of breast tumor cells (Chen and Bourguignon, 2014). Treatment strategies based on targeting CD44 decreases the glucose consumption and ATP by the cancerous cells. The expression of CD44 might be associated with B-catenin and AKT pathways, so CD44 expression could be regulated by inhibiting either pathway or both. CD44 cells also play a major role in metastatic cascade in various carcinomas. Metastatic cascade is regulated by epidermal growth factor receptor (EGFR)/ErbB1 and ErbB2/Her 2 receptors whose overexpression form the most aggressive kind of breast cancer. Ras/SOS is activated by the interactions of HA/other ligands with CD44, and regulates the growth and invasion cascade (Nam et al., 2016). In a study, the CD44 role in Smad-dependent invasion was reported along with its effect on TGFb receptor 1 and 2 triggering CD44 binding. The activation of Rho ATPase by CD44 enhances the cytoskeletal transformation and invasion, while other pathways like PI3K-AKT and MAPK-Ras enhances tumor cell growth, survival, and invasion (Bourguignon, 2019).

## CD44 Engineered Nanocarriers for Cancer Therapy

**FIGURE 4:**
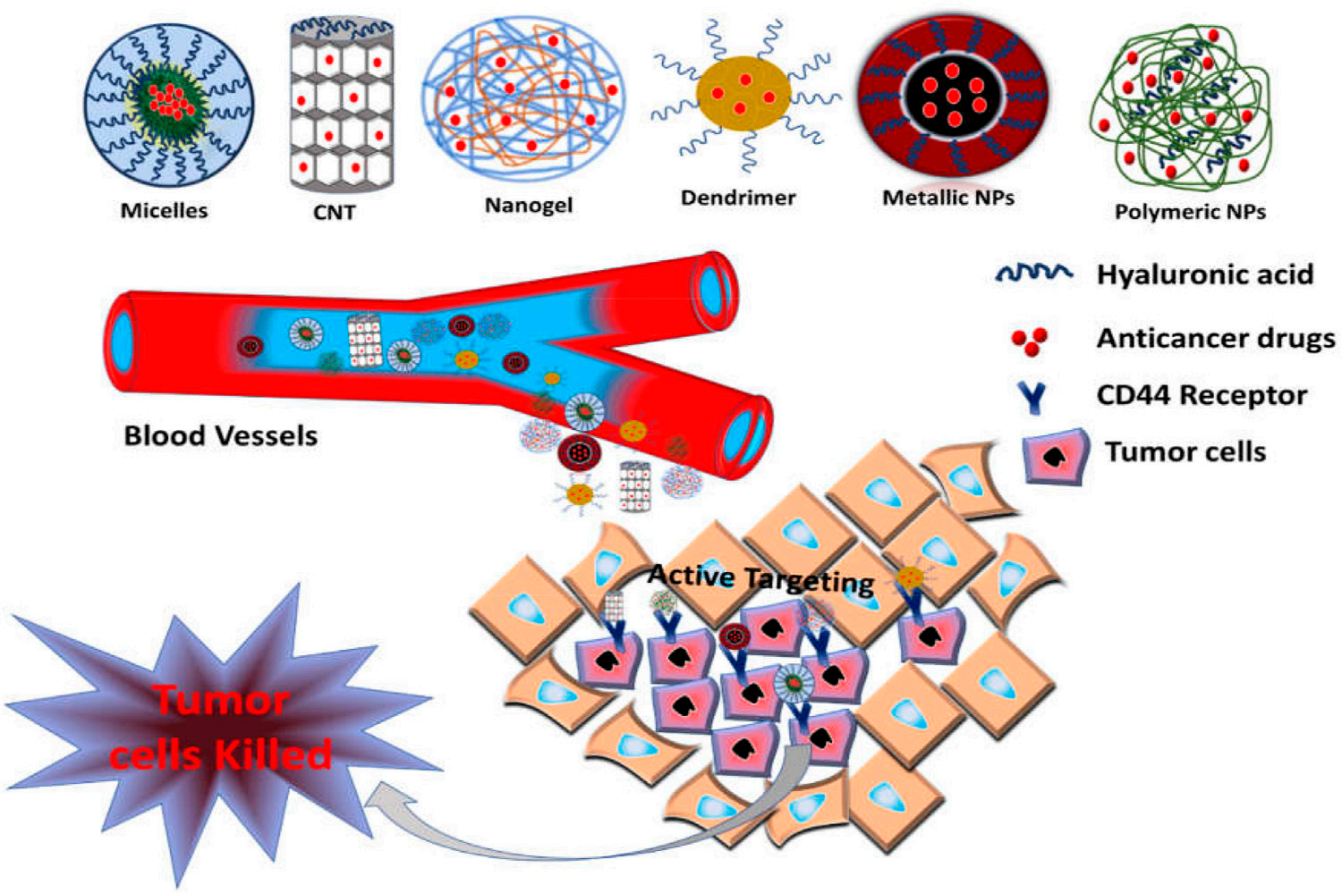
Schematic illustration of CD44-targeted delivery of anticancer drugs.

Owing to good biodegradability and biocompatibility, NPs targeting the CD44 receptor could deliver the therapeutic agent directly to the cell in a controlled manner, sparing its effect on normal cells (Lai et al., 2021). Such a nano-drug delivery system also offers modification with attachment on the surface that could elevate its uptake and prompt apoptosis. Nanocarriers also offer multiple advantages, for instance, ease of production, high drug loading capacity, improved solubility and bioavailability, improved distribution of drug inside the body, enhanced permeability to physiological barriers, and decreased drug toxicity (Khurana et al., 2017) (Figure 4).

### CD44 Engineered Liposomes for Cancer Therapy

**FIGURE 5:**
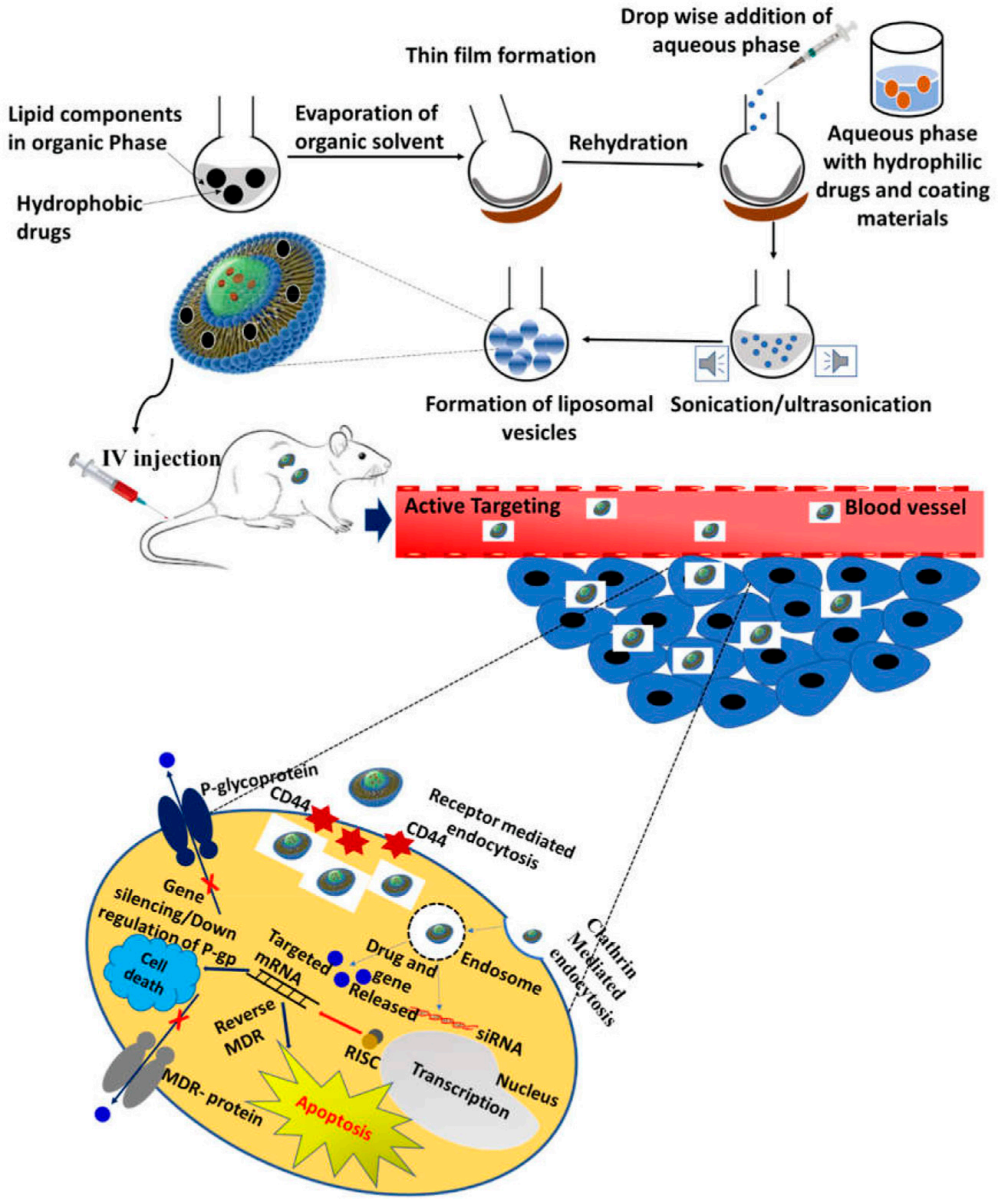
Schematic illustration of liposome-based CD44-targeted delivery of anticancer drugs/genes.

Liposomes are spherical vesicles composed of phospholipid bilayers with an enclosed aqueous core. The lipid bilayer may be natural or synthetic, single bilayer vesicles are known as unilamellar vesicles; multiple bilayer vesicles are termed as multilamellar vesicles. Liposomes can encapsulate both hydrophilic and hydrophobic drug molecules into their bilayers. They may vary in size, surface, composition, and methods of preparations. Nowadays, the liposome is the most commonly used model nanocarrier for the delivery of numerous bioactive molecules such as drugs, vaccines, cosmetics, genes, nutraceuticals, and various combination therapies (Deshpande et al., 2013; Torchilin, 2005). It is also a preferred carrier for targeting delivery, especially for anticancer drugs, that shows various advantages over the conventional system of which targeting delivery is among the most considered (Figure 5). It helps in reduction of side effects of many chemotherapeutics agents by preventing them from distribution to non-targeted sites. Liposomes can alter the pharmacokinetics of drug carriers by their surface functionalization (Sharma et al., 2006). They can easily be functionalized with the various targeting moieties, such as polysaccharides [folate (FA), hyaluronic acid (HA), chitosan (CH), etc.], peptides, genes, and antibodies (Accardo and Morelli, 2015; Ruttala et al., 2017; Sofou and Sgouros, 2008; Zylberberg et al., 2017) for delivering chemotherapeutics to specific tumor cells. Here, we have discussed liposome-based nanocarriers targeting CD44 tumor cells by using the most potent ligand hyaluronic acid (HA) to bind with target CD44 cells (Table 1). Song et al. had designed hyaluronic acid (HA)-modified liposomes for targeting CD44 cells expressed on breast cancer and codelivery of paclitaxel (PTX) and doxorubicin (DOX). Firstly, paclitaxel, hydrogenated lecithin, and cholesterol in a particular ratio was dissolved into a solution of methanol: chloroform at a ratio of 1:3. The organic solvent was then evaporated by a rotary evaporator to form a thin film, the overnight-dried film was hydrated with ammonium solution at 47°C for 1 h, and subsequently sonicated to achieve the desired vesicle size. DOX was also loaded into the liposomes by the thin film hydration method with minor changes. Both DOX and paclitaxel was loaded into the HA-modified liposomes using the post-insertion method with small variation Briefly, a small amount of cholesterol-conjugated HA was incubated with the above solutions containing DOX and paclitaxel-loaded liposomes for half an hour at the temperature of 50°C. The prepared formulation showed a particle size range between 90 and 130 nm and PDI <0.25. HA conjugation with liposome increased the particle size of the liposome, and also changed its surface charge. The negative charge of unmodified liposomes changed to a positive charge due to the carboxy group of HA. HA conjugation of liposomes acts as a PEG molecule in circulation, which increases the blood retention time by escaping clearance *via* the RES system. Encapsulation efficiency (EE) of HA-decorated liposomes (HA-D-P-liposome) was 93.6 % and 70.4% for DOX and paclitaxel, respectively. TEM images exhibited spherical shape and uniform size distribution particles of HA-D-P-liposome. The amount of cholesterol-HA into the HA-modified lipid was determined by the carbazole method, that was found to be 9.5% mol of the total lipids. *In vitro* comparative cellular uptake of HA-DOX-liposome, DOX-liposome, and free DOX was also evaluated using MCF-7 (CD44^+^ receptors) and HepG2 (CD44^−^ receptor) cell lines through confocal microscopic imaging. Fluorescent intensity of HA-DOX-liposome was higher than the free DOX and DOX-liposome treated in MCF-7 cells, whereas HepG2 cells showed comparatively less fluorescence intensity. This is due to overexpression of CD44 receptor on MCF-7 cells, which enhanced the receptor ligand (HA)-mediated transport of HA-DOX-liposome (active targeting) whereas in HepG2 cells due to low expression of CD44 cells, the ligand (HA)-mediated transport of HA-DOX-liposome was hampered, however, free DOX and DOX-liposome traversed by passive diffusion. Scientists also demonstrated the high uptake of HA-DOX-liposome on MCF-7 cell lines which were majorly due to caveolae, clathrin-mediated endocytosis, and the ligand housing effect. MCF-7 cell lines pre-treated with the free HA significantly decreased the HA-DOX-liposome cellular uptake. This demonstrated the ligand binding capability of HA towards CD44. A cytotoxicity study revealed that both HA-DOX-liposome and HA-PTX-liposome showed higher cytotoxicity than free and unmodified drug-loaded liposomes. The IC50 value for drug-loaded HA-modified liposomes was lower than free or drug-loaded unmodified liposomes, the reason for such an effect may be due to high cellular uptake, control release, and drug resistance. As compared to individual drug treatment, co-loaded treatment had superior cytotoxicity. The IC50 value for DOX/PTX-liposome and HA-D/P-liposome was 0.51 and 0.14, respectively, HA-D/P-liposome showed the overall lowest IC50 value among the formulations, illustrating that the HA-modified codelivery system had a synergistic cytotoxic effect (Song et al., 2019). In another similar study, Mahira et al. targeted the CD44 receptor overexpressed on prostate cancer stem cells (CSCs) using anionic polysaccharide-coated cationic liposomes. In this study, researchers had co-loaded cabazitaxel (CBX) and silibinin (SIL) in liposomes to evaluate the anticancer effect against CSCs. Liposomes were prepared by dissolving cationic phospholipid N-[1-(2,3-Dioleoyloxy) propyl] N, N, N-trimethyl ammonium chloride (DOTAP), cholesterol, CBX, and SIL in common solvent (ethanol). The mixture was added dropwise into the aqueous solution of TPGS under continuous stirring followed by solvent evaporation. For the preparation of HA-coated liposomes, HA was dissolved into the aqueous solution with TPGS followed by dropwise addition to the organic phase. Researchers selected HA-wrapped liposomes with 10% w/w drug loading, a 1/5 phase volume ratio, and 0.1% HA concentration from the diverse batches due to their small size and PDI. Anionic polysaccharide with a concentration of 0.1 mg/ml showed optimum results which masked the cationic surface charge of liposomes, resulting in reduced toxicity of liposomes due to cationic surface charge and finally helped to target tumor cells. Comparative cellular reduced CD44 cells were studied for active HA receptor targeting on PC-3 and DU-145 prostate cancer cells. HA-coated liposomes showed 4.03 times more CD44 population suppression as compared to other formulations. Obtained results proved the concept of active targeting of CD44 cells, which enhanced the therapeutic benefits and reduced non-specific distribution leading to reduced toxicity. Cytotoxicity results showed IC50 values of 78 ng/ml, 46 μg/ml, and 23 ng/ml, respectively for CS-Sol, CS-liposome and HA-CS-LP (C and S-loaded HA-modified liposomes) in PC-3 cells, whereas, 17.6 ng/ml, 160 ng/ml, 29 ng/ml, respectively in DU-145 cells for HCS-LP, CS-LP, and CS-sol. HCS-LP comparatively exhibited efficient cell growth inhibition with the lowest IC50 value in both cell lines. These outcomes again denoted that HA coating encouraged efficiency of combined drug treatments. Cell cycle inhibition results revealed that CS-loaded HA-liposome showed strong knockdown of PC cells by arresting the cell cycle at the G2/M phase due to the increment in the intracellular concentration of both drugs. The synergistic therapeutic effect of C and S by HA-coated liposomes targeting the CD44 receptor against CSCs was also concluded from this study (Mahira et al., 2019). Fan and co-workers reported a new combination of nanocomplex for co-delivery of gemcitabine (GEM) and docetaxel (DTX) against triple negative breast cancer. GEM was linked with HA by esterification. This HA-modified nanocarrier had great potency for active targeting of overexpressed CD44 receptors on MDA-MB-231 cell lines. DTX-loaded liposome (DTX-LP) was prepared by usual thin film hydration methods. Finally, the GEM/DTX coloaded nanocomplex was obtained by dropwise addition of DTX-LP into the HA-GEM solution under continuous stirring at 4°C. Prepared NCs exhibited suitable characteristics with a size range <200 nm, PDI <0.2, zetapotential −31.1 mV, sufficient drug loading (GEM 9.3% and DTX 3.1%), and stimuli-responsive release characteristics. The prepared formulation showed higher *in vitro* cellular uptake in MDA-MB-231 cell lines than MCF-7 cell lines. A HA receptor block assay was also performed to confirm receptor-meditated endocytosis, that revealed that the fluorescence intensity for HA/C6-CL NCs decreased drastically in MDA-MB-231 cell lines while there was no change in fluorescence intensity in MCF-7 cell lines. Thus, it was concluded that the CD44 receptor-mediated endocytosis is strongly receptor-dependent. Moreover, higher cellular uptake in the MDA-MB-231 cells than MCF-7 cells was due to its overexpression of CD44 receptors. An *in vitro* cytotoxicity study revealed that among the different formulations, cells treated with a nanocomplex exhibited increased cytotoxicity compared to free drugs showing an IC50 value of 0.67 ng/ml, which was 0.14-fold more than the free drug. This further confirms the successful uptake of the nanocomplex mediated by the CD44 tumor homing effect with successful endosomal and lysosomal escape. Moreover, the minimum inhibitory concentration and cell apoptosis confirms that both drugs could induce synergistic cytotoxic effect in combination with better targeting potential. The nanocomplex also elevated the S phase/cell cycle inhibition capability and modification of CDA and dCK balance *via* decreasing mRNA expression of CDA (Fan et al., 2017). In another study, Alshaer et al*.* evaluated the targeting effect of aptamer-mediated PEGylated liposomes against CD44 receptor positive cells (A549 and MDA-MB-231 cell). The PEGylated liposomes were prepared by the thin film evaporation method *via* a thiol-maleimide click reaction. Here, cholesterol, and DSPE-PEG (2000)- Mal were dissolved in common solvent (chloroform). The thin film was obtained after evaporation of chloroform followed by hydration with PBS solution. Rhodamine-linked liposomes were used as the blank control. PEG-Mal-liposomes was conjugated with an anti-CD44 2′-F-pyrimidineRNA aptamer by the thiol-maleimide click chemistry reaction. The aptamer selectively binds to CD44-expressing tumor cells. Conjugation of the aptamer with the liposome was confirmed by change in size, zeta potential, and gel electrophoresis. The hydrodynamic size of Mal-Lip increased from 129 ± 5 nm to 140 ± 6 nm after conjugation with the aptamer. Similarly, zetapotential shifted from −17.5 ± 0.9 mV for Mal-Lip to −31.0 ± 2.3 mV. An increase in size and zeta potential confirmed successful binding of the aptamer to liposomes. The binding affinity of the aptamer-liposome complex (Kd = 6.2 ± 1.6 nM) was comparatively better than the free aptamer (Kd = 21.5 ± 3.3 nM). Moreover, the free aptamer exhibited higher binding saturation than the aptamer-liposome complex which blanketed a large portion of beads causing steric hindrance of the residual free binding sites. This was further confirmed by *in vitro* flow cytometry and a confocal microscopy assay, showing higher uptake of the aptamer-liposome complex than rhodamine-loaded liposomes. However, in CD44 negative cells (NIH/3T3), there were no significant change in fluorescent intensity after treatment with targeted and non-targeted liposomes confirming the affinity of aptamer to target specific cells without affecting the normal human cellular structure (Alshaer et al., 2015). With the surge in oncology-based research, experts are studying the tumor microenvironment (TME) to get familiar with the reason of tumor progression, metastasis, and drug resistance. The TME is composed of immune cells, fibroblasts, and majorly the extracellular matrix (ECM). Any alteration in the ECM affects cell progression and metastasis by regulating the TME. Matrix metalloproteinases degrades the ECM, for instance fibronectins, gelatin, and collagen causing a surge in tumor progression. To overcome such a hurdle, Lv et al. designed self-assembled hybrid nanoparticles (H_Np) using the PTX-HA prodrug and Matt-loaded lysolipid-based thermosensitive liposomes for targeting TME and CD44 positive metastatic breast carcinoma. Matt is an enzyme inhibitor that suppresses the progression of tumors by affecting their metastasis. The formed (H_Np) exhibited a particle size, PDI, and surface potential of 95.60 ± 1.10 nm, 0.24 ± 0.01, and −1.62 ± 1.25 mV, respectively. H_Np released its payload into the extracellular environments triggered by hyperthermia. The thermosensitive dual targeted H_Np was rapidly up taken by 4T1 tumor cells due to its high binding affinity of CD44 towards HA with 1.6-fold tumor accumulation, 10-fold tumor growth inhibition activity, and 10-fold angiogenesis as compared to non-targeted preparation.

**TABLE 1:** Summary of liposome-targeted CD44 cells for cancer therapy.

Types of liposomes	Therapeutic agents	Types of cancer	X′ cell lines	Animal model	References
HA-modified liposomes	DOX and PTX	Breast cancer	MCF-7 and HepG2	—	Song et al. (2019)
Cationic liposomes	Cabazitaxel and silibinin	Prostate cancer	PC-3 and DU-145	—	Mahira et al. (2019)
Cationic liposomes	Gemcitabine and docetaxel	TNBC	MCF-7 and MDA-MB-231	Mice	Fan et al. (2017)
PEGylated liposomes	Anti-CD44 2′-F pyrimidine RNA aptamer	Lung and breast	A549 and MDA-MB-231	—	Alshaer et al. (2015)
HNPs	Paclitaxel	Metastatic breast cancer	4T1, MCF-7, A549, and NIH/3T3 cells	Mice	Lv et al. (2018)
PEGylated liposomes	Stigmasterol and DOX	Breast cancer	MCF-7 and MDA-MB-231	Mice	Gautam et al. (2020)
Polymersomes (PEG-PCL)	DOX	Breast cancer	MCF-7 and 4T1	Mice	Shahriari et al. (2019)

Notably, H_Np inhibited >5-fold expression and activity of MMP, and also blocked activation of fibroblasts *via* the suppressing expression of TGF-β and downregulated extracellular matrix degradation. Finally, PTX-HA active compound and H_Np were easily self-assembled into a multifunctional nanocarrier for simultaneously targeting TME and CD44 cells (Lv et al., 2018). Thus, this study offered a proof of concept that self-assemble nanoparticles dual targeting TME and CD44 cancer cells using PTX-HA and Matt with H_Np was efficient for the treatment of metastatic breast cancer. Gautam et al. had fabricated HA-modified PEGylated phyto-liposome for the targeted delivery of stigmasterol (STS) and DOX for breast cancer treatment. Optimized formulation exhibited acceptable particle size and surface characteristics. After coating with HA, surface charge changed from +17.1 ± 0.8 mV to −9.5 ± 0.2, while the particle size and PDI were found to be 173.9 ± 2.4 nm and 0.26 ± 0.01, respectively. These parameters confirmed that the drug-loaded liposomes (HA-STS-DOX-Lip) were coated with HA. The targeted liposomes exhibited a sustained release profile over a period of 48 h. *In vitro* confocal microscopy displayed a significantly higher uptake of HA-STS-DOX-Lip in CD44-overexpressed MDA-MB-231 cells than MCF-7, this might be due to CD44 receptor-mediated endocytosis, where HA interacted with membrane-bound CD44 protein. At the same condition, uptake of HA-STS-DOX-Lip into MDA-MB-231 cells declined in the presence of free HA, which means free HA competes with HA-STS-DOX-Lip for CD44 binding. HA-STS-DOX-Lip also exhibited deep penetration into the nucleus showing higher apoptosis in MDA-MB-231 cells as compared to free DOX and STS. Accumulation of HA-STS-DOX-Lip was observed into the tumor site only which might be due to CD44 homing and ERP effect. The *in vivo* anticancer study revealed significant higher efficacy of targeted preparation than free DOX, STS, and STS-DOX-lip showing much less toxicity than the free drug (Gautam et al., 2020). In another study, Shahriari et al. had designed a novel polymer-based formulation for CD44-targeted delivery of DOX. In this study, HA was used as a ligand to target CD44 cells of breast cancer. Initially carboxylated polycaprolactone (PCL-COOH) was obtained by dissolving and treating 1, 4 dioxane with succinic anhydride, DMAP, and triethylamine. The PCL was activated with the treatment of EDC or NHS. NHS-activated PCL was coupled with aminated HA by dissolving in DMF and DIPEA under gentle stirring. HA-PCL was formed by amide linkage between HA and PCL functional groups. The nanoprecipitation method was used to prepare blank PCL-HA polymersome and DOX-loaded HA-PCL polymersome. Moreover, DOX was loaded into the PEG-PCL NPs by the double emulsification method. *In vitro* comparative cellular uptake along with cancer targeting results of formulations such as PCL-HA-DOX, PEGylated-PCL-DOX along with free DOX in the MCF-7 and 4T1 cells revealed that PCL-HA-DOX NPs exhibited higher cellular uptake than PEGylated-PCL-DOX and free DOX in both cells. A decrease in uptake of PCL-HA-DOX NPs was observed when cells were pre-treated with HA. HA bound and occupied the CD44 receptors overexpressed in cancer cells causing decreased cellular uptake of PCL-HA-DOX. Due to surface modification of nanoparticles by HA, *in vivo* antitumor efficacy, bio-distribution parameters, and tumor tissue necrosis ability were elevated (Shahriari et al., 2019).

### CD44-Engineered Nanoparticles for Cancer Therapy

Nanoparticles may be defined as a small particle ranging between 1–100 nm in size. They show notably physical as well as chemical properties different from their parents’ larger materials. For example, reduction in size of any materials to nano size range increases the surface area, enabling a higher chance of interaction with the surrounding materials affecting their reactivity. Such an alteration in physicochemical properties of NPs also influences the alteration/change in electric, magnetic, electric, chemical, and optical properties (Jeevanandam et al., 2018).

Application of nanoparticles in the field of medicine is increasing day by day. NP-based drug delivery systems are a focused research area in cancer therapy and other prominent and emerging diseases. NPs can influence the pharmacokinetics, distribution, and accumulation of therapeutic moiety into the specific site which reduces toxicity. In comparison to conventional drug delivery for cancer theragnostics, nanoparticle-based therapy provided better results. Nanoparticles improved several physicochemical properties of many therapeutic drug molecules, increased aqueous solubility, and modified the release pattern providing significant accumulation of the drug at the site of the tumor (Rizvi and Saleh, 2018).

Numerous methods are available for the manufacturing of NPs. However, most NPs are prepared by two methods namely 1) the top-down method (chemical etching, mechanical milling, sputtering, electro explosion, and laser ablation) and 2) bottom-up method (solvent evaporation, solvent precipitation, laser photolysis, atomic and molecular condensation, and biological synthesis by bacteria, virus, yeast, or fungi). Due to their nanometric size, NPs possess several unique advantages such as passive tumor targeting of incorporated therapeutic molecules, which accumulate passively into the leaky vasculature of tumor cells through the ERP effect. Their small size of <20 nm can pass through blood vessel walls, and it allows for IV, IM, and SC administration, these injections reduce irritation reactions at injection sites due to their small size. To deliver efficient cargos at specific sites in a controlled way, NPs are manufactured in a defined way using specific materials. There are many types of NPs based on material construct which are mainly carbon-based NPs (CNT), metallic NPs (Au, Fe2O3), ceramic NPs, polymeric NPs, and lipid-based NPs. Tailoring nanocarriers suitable for combination therapy is challenging and requires multiple refinement processes. NPs can be decorated as per need (Wolfbeis, 2015; Khan et al., 2019) (Table 2).

**TABLE 2:** Outline of nanoparticle-targeted CD44 cells against cancer.

Polymers used to prepare NPs	Therapeutic agents	Types of study (*in vitro*/*in vivo*)	Cancer types	Cell lines	Animal model	References
PLGA	SLM and PTX	Both	Mammary gland	MCF-7	SD rats	Muntimadugu et al. (2016)
PLGA	TTQ	*In vitro*	Multiple	MCF-7, MDA-MB-231, and MiaPaca-2	——	Saneja et al. (2017)
Chitosan, chondroitin SO4, and PLGA	HCPT	Both	Colon cancer	C-26	Mice	Liu D et al. (2019)
HA	DOX and cisplatin	Both	Mammary carcinoma	4T1 and NIH-3T3	Female BALB/c mice	Yu et al. (2020)
PLL	PTX and GEM	Both	Biliary	HuCCT1 and SCK	BALB/c nude mice	Noh et al. (2015)
LbL lipid NPs	Berberine and rapamycin	Both	Lung	A549	Male Albino mice	Kabary et al. (2018)
PLGA	Bromelain	Both	Mammary gland	HEK293, MCF-7, A549	Swiss albino mice	Bhatnagar et al. (2016)
BSA	ATRA	Both	Lung cancer	B16F10 cells	Mouse	Li et al. (2018)
SLN	DTX	Both	Skin	B16F10 CD44+/αvβ3+	C57BL/6 mice	Shi et al. (2016)
PAMAM	DTX and alendronate	Both	Lung	RAW264.7	Nude mice	Bai et al. (2019)
HA	MTX and alendronate	*In vitro*	Cervical Carcinoma	HeLa, A549, and HUVEC cells	-	Gao et al. (2021)
PEI and PEG	Daunorubicin	Both	AML	HL60 cells	BALB/c mice	Bai et al. (2021)
MSNs	MTX	*In vitro* and in silico	Mammary gland	MDA-MB-231 and MCF-7	-	Sargazi et al. (2018)
MSNs	Au	Both	Cervical cancer	HeLa and U87MG cells	Mice	Li et al. (2015)
MSNs	FI	Both	Cervical cancer	HeLa and U87MG cells	Mice	Li et al. (2014)
MSNs and PEI	OXL	Both	Colon cancer	HT-29	BALB/c nude mice	Yang et al. (2019)
MSNs	-	*In vitro*	Cervical	HeLa cells	—	Nairi et al. (2018)
MSNs	-	*In vitro*	Breast	MDA-MB-231 and A2780	—	Ricci et al. (2018)
MAL and PEG	DTX	Both	Breast	MCF-7	BALB/c nude mice	Lee H et al. (2020)
HA ceramide FA	Dox	Both	Ovarian	SKOV-3	BALB/c nude mice	Lee et al. (2016b)
PEG-gelatin	Epigallocatechin-3-gallate	Both	Prostate	PC3	Nude mice	Huang et al. (2016)
Adipic dihydrazide	Curcumin	Both	Mammary gland	4T1 and MCF-7	Mice	Lai et al. (2021)

#### Polymeric Nanoparticles

There are lots of synthetic or natural polymers that can be used as NP constructs for the therapeutic delivery of anticancer molecules; most used polymers are biodegradable. Most of these polymers can easily conjugate with hyaluronic acid and other targeting ligands to achieve targeted drug delivery against different types of cancer (Begines et al., 2020).

Muntimadugu et al*.* designed and developed a poly (lactic-co-glycolic acid) (PLGA) nanoparticle for the CD44-targeted delivery of anticancer drug molecules, salinomycin (SLM) to kill cancer stem cells (CSCs) and paclitaxel to weaken cancerous cells. The PLGA nanoparticle was prepared by the emulsion solvent diffusion method. To carry out the cellular uptake study, FITC-loaded NPs were also prepared. The prepared targeted and non-targeted nanoparticles were characterized using DLS to measure the size and PDI. The wrapped SLM-NPs with HA showed concentration-dependent charge inversion. The entrapment efficiency increased from 63.5 ± 1.2% to 71.2 ± 3.4% after coating with HA, which is due to more surface availability due to the coating of nanoparticles. *In vitro* cytotoxicity into the non-sorted, non-isolated MCF-7 cells showed concentration-dependent cytotoxicity. A 100 μM concentration of SLM-HA-NP-treated cells exhibited poor cell viability of 6.9 due to CD44 receptor-mediated cellular uptake. The IC50 value also confirmed the idea of receptor-mediated cellular uptake by showing a 4.1-fold lower IC50 value of SLM-FA-NPs than SLM. FITC-HA-NPs exhibited 1.5 times higher uptake than non-targeted FITC-NPs. The % cell inhibition was obtained in the following order PTX < SLM < SLM/PTX, additionally a significant decrease in CD44 + cell count was observed in SLM-HA-NPs, confirming active targeting ability of HA-coated NPs (Muntimadugu et al., 2016). Saneja et al. used PEGylated PLGA-HA for the targeted delivery of a thio-tetrazoly analogue of a clinical candidate IC87114 (TTQ). HA-modified PEGylated PLGA nanoparticles exhibited significant greater cellular internalization and cell cytotoxicity as compared to HA-non-modified PEGylated PLGA nanoparticles. Cellular uptake and cytotoxicity results of PLGA-PEG-HA NPs were found in the following order MCF-7 >MDA-MB-231 >MiaPaca-2 cells, these differences in the cellular internalization in different cell lines depended on the level of CD44 receptor expression. The level of expression of CD44 receptors in the following cell lines MCF-7, MDA-MB-231, and MiaPaca-2 were low, medium, and large. The therapeutic benefit of the prepared targeted delivery system was based on binding of the HA ligand on to the CD44 overexpressed receptor on the tumor cells (Saneja et al., 2017).

In another study, Liu et al. prepared chitosan (CN) and chondroitin sulfate (CS)-modified PLGA NPs for the targeted delivery of an anticancer drug (10-hydroxy camptothecin) (HCPT). Tirella et al. reported CS had a hyaluronic acid-like structure, so can be identified by the HA receptor (CD44) (Wang et al., 2016; Tirella et al., 2019). Prepared NPs were denoted as HCPT-CN-PLGA NP and HCPT-CS-CN-PLGA NP. *In vitro* cellular uptake results into the Colon-26 cells exhibited around a 40% higher uptake of Cy5-CS-CN-PLGA NP than Cy5-CN-PLGA NP, due to the overexpression of the CD44 receptor on C26 cells, which is due to the affinity towards the CS ligand. Numbers of tumor nodules after treatment of tumor-induced mice reduced by both HCPT-CS-CN-PLGA NP and HCPT-CN-PLGA NP formulations, the model group exhibited 23.6 tumor modules. HCPT-CS-CN-PLGA NP dramatically reduced TNF-α and IL-1β levels in tumor tissues than HCPT-CN-PLGA NP. Such results are attributed to surface modifications by CS which helped the ingestion of the formulation into tumor cells resulting in cell death (Liu D. et al., 2019). Lee et al*.* designed a chondroitin sulfate A and MAL-based nanostructure which enhanced tumor targeting and penetration due to selective CD44 receptor binding and selective binding of MAL to the thiol group of blood components and cellular membrane (Lee H. et al., 2020). Huang et al. had successfully delivered a polyphenol extract of green tea epigallocatechin-3-gallate (EGCG) by HA-PEG-gelatin NPs to inhibit MMP associated with tumor metastasis and invasion against prostate cancer. In this study, EGCG-loaded HA-PEG-gelatin-based NPs showed inhibition of tumor metastasis, cell cycle arrest at the G2/M phase, and inhibited the growth of prostate cancer. The *in vivo* results also showed CD44 receptor-based anticancer activity against prostate cancer (Huang et al., 2016).

Yu and co-workers evaluated CD44-targeted antitumor activity of HA-modified DOX-CDDP (cis-diamminedichloroplatinum, cisplatin) NPs. *In vitro* cellular uptake was evaluated by flow cytometry and CLSM against 3T3 and 4T1 cells incubated with free drug and HA-coated DOX-CDDP for 6 h cells. HA-coated DOX-CDDP-treated 4T1 (CD44 +) cells displayed significantly higher internalization than the group treated with free drugs and pre-treated with HA, while in 3T3 (CD44 low expression) cells, internalization of free drug was higher than other formulations. These results prove the uptake of nanoparticles is mediated by the presence of CD44 in cells. *In vitro* cytotoxicity of the HA-coated DOX-CDDP-treated group showed significant higher cytotoxicity against 4T1 cells than other groups. An *in vivo* biodistribution study showed 2.1 times higher fluorescence intensity in the tumor cell treated with HA-coated DOX-CDDP than the free drug group 12 h after injection. Histopathological analysis revealed a 56.3% ± 5.8% tissue necrosis area for the HA-coated DOX-CDDP-treated group which was higher than the free drugs (35.65 ± 6.5%) and non-treated groups (1.5% ± 0.8%). Conclusively, for reducing side effects, targeting drugs specifically to receptor overexpressed cells can potentiate therapeutic advantage (Yu et al., 2020). In another study, *Noh and co-workers* co-delivered two anticancer drugs namely PTX and GEM *via* a HA-modified poly (L-lysine) carboxylate nanocarrier. The study revealed, *in vitro* and *in vivo*, that the prepared PTX/GEM-loaded HA-conjugated treatment exhibited significantly higher anticancer effect in HuCCT1 (high level of CD44 receptor expression) cells than that of SCK (high level of CD44 receptor expression). Both drugs synergistically induced apoptosis against HuCCT1 cells. These results suggested HA-dependent CD44 receptor-mediated endocytosis playing a crucial role in cancer apoptosis (Noh et al., 2015). Kabarya et al. co-delivered berberine (BER) and rapamycin (RAP) by designing layer by layer NPs functionalized using tumor targeting polymers lactoferrin (LF) and HA. *In vitro* cytotoxicity results showed that LBL NPs coated with polymers had significantly increased cytotoxicity against A549 cell lines than non-coated LBL, *via* increased cellular uptake by specific binding with overexpressed CD44 receptors. HA-based CD44 receptor-mediated endocytosis was also verified *in vivo*, both drugs loaded with LBL-HA exhibited an 88.09% average number of microscopic tumor foci reduction which was remarkably higher than free drugs, moreover BER-RAP-loaded LBL-HA reduced VEGF (tumor growth factor) 3.1 times compared to positive control (Kabary et al., 2018).

Bhatnagar et al. used a HA-grafted PLGA copolymer for the targeted delivery of anticancer phytoconstituents bromelain (BL). The BL-loaded copolymer (BL-HA-PLGA NPs) showed a size ranging between 140 nm and 281 nm. BL-HA-PLGA NPs exhibited significant higher cell cytotoxicity than free BL or BL-PLGA NPs. BL-HA-PLGA NPs also showed significant reduction in IC50 value than free BL and BL-PLGA NPs, which clearly indicates enhanced capability of BL-HA-PLGA NPs to kill tumor cells more effectively. Remarkably, cytotoxic results are mediated by active and passive targeting by BL-HA-PLGA. Fluorescence (F)-labeled BL-HA-PLGA NPs showed 3.5 times higher cellular uptake than PLGA-NPs and free drugs with higher accumulation in MCF-7 cell than A549 cells. This prominent difference was contributed *via* the expression level of CD44 receptor on tumor cells. HA receptor-mediated cellular internalization was also confirmed in the presence/absence of free FA. *In vivo* cell anticancer activity of free BL, BL-PLGA-NPs, BL-HA-PLGA NPs, and control group showed 90–95% cell viability. Non-targeted BL-PLGA NPs reduced viability to 40 ± 3.75%, whereas BL-HA-PLGA NPs reduced cancer viability to 11 ± 2.18% which was significantly better than free BL and BL-PLGA NPs. Targeted nanoparticles showed better anti-cancer activity with improved survival rate in mice than non-targeted preparations (Bhatnagar et al., 2016).

Similarly, Li et al. used the naturally occurring derivative of retinoic acid called all trans retinoic acid (ATRA) and successfully loaded it into the HA-modified albumin nanoparticles. The prepared cationic albumin nanoparticle showed 93% encapsulation efficiency and 8.37% drug loading for ATRA, with spherical shape, and uniform size and with a highly stable nanoparticle. HA-coated nanoparticles exhibited significantly higher uptake into the CD44 ^+^ B16F10 cells than non-targeted NPs which is due to higher affinity of the HA CD44 + receptor. The targeted nanoparticles showed a decrease in tumor size compared to the non-targeted preparation explaining the effect of ligand-mediated endocytosis (Li et al., 2018).

Shi et al*.* designed and developed a novel dual targeting system to enhance the targeting efficiency of chemotherapeutic therapy. A dual targeting system conjugated HA (ligand for CD44 receptor) and tertaiodo-thyroacetic acid (tetrac) (ligand for αvβ3) to a solid lipid nanoparticle for synergistic active targeted delivery of DTX. Prepared NPs were denoted as SLN/DTX, Te-SLNs/DTX, HA-SLNs/DTX, and TeHA-SLN/DTX. TeHA-SLN/DTX exhibited DTX >91.6% encapsulation efficiency showing toroid morphology. Results of *in vivo* imaging and vessel distribution tests showed significantly higher synergistic active targeting efficiency for TeHA-SLNs toward αvβ3 and CD44 receptors than that for ERP-mediated passive targeting. These obtained results strongly recommended that an enhancement in the efficacy TeHA-SLNs/DTX against tumor cells was strongly reliant on the specific interactions between TeHA ligands and CD44/αvβ3 receptors, both ligands did not inhibit the interactions with their particular target but exhibited a synergistic dual active targeting effect (Shi et al., 2016). In another study, by Bai et al*.,* the CD44 receptor of osteoclasts and bone metastatic tumor cells was effectively targeted and inhibited osteoclast and bone metastasis of lung cancer by using a DTX-loaded alendronate (ALN)-HA-PAMAM (AHP) nanocarrier. Scientists investigated therapeutic effectiveness of this formulation both *in vitro* and *in vivo*, and reported its effectiveness in both studies, in which CD44 receptor-mediated endocytosis played a significant role (Bai et al., 2019). Recently, in another study, Gao and co-worker investigated the *in vitro*/*in vivo* antitumor activity of dual receptor-targeted HA-modified alendronate-methotrexate (HA-ALN-MTX-NPs) and they found that HA-ALN-MTX-NPs had a significantly higher cytotoxic effect against A549 (overexpressed CD44 receptor cells) than that of HUVECs (normal tissue) cells. This is due to selective uptake by tumor cells through specific binding of HA with the CD44 receptor on tumor cells (Gao et al., 2021).

Most recently, Bai et al. reported a pH-responsive, dual targeting nano system for the treatment of acute myeloid leukemia (AML). They targeted CXCR4 and CD44 overexpressed receptor on AML. Here, PEG-grafted PEI *via* benzoic imine bond decorated with Prussian blue NPs (PBNPs) were prepared, which was further functionalized with CXCR4 targeting peptide E5 and HA. Daunorubicin (DNR)-loaded dual-targeted PBNPs efficiently escaped endosomal uptake and increased circulation time in the physiological condition. Dual-targeted PBNPs also showed significantly enhanced cellular uptake and anti-HL60 cell effect leading to reduced tumor migration than mono-targeted PBNPs. *In vivo* results of DNR-loaded dual-targeted PBNPs exhibited significantly improved anticancer and anti-metastatic results against AML treatment (Bai et al., 2021). Lee et al. synthesized unique types of dual-targeting nanocarriers consisting of hyaluronic acid and ceramide folic acid (HACE-FA) in which the -COOH group of FA was reacted with the -OH group of HA to bind *via* the ester bond. DOX was loaded into the HACE-FA and was successfully delivered against SKOV-3 cells (CD44 and FR positive cells). Accumulation of HACE-FA NPs was significantly higher than HACE NPs, while conjugation of FA to HACE NPs enhanced uptake efficiency around 67%. *In vivo* NIRF imaging also confirmed the dual targetability of DOX-loaded HACE-FA NPs by showing 3.81-fold higher fluorescence intensity in the tumor group than that of DOX-loaded HACE NPs. HACE-FA NPs were primarily accumulated in the tumor site and exhibited around a 4.82-fold higher fluorescent signal than HACE-FA NPs. Finally, the researcher suggested that DOX-loaded HACE-FA NPs were synergistic in *in vitro* and *in vivo* therapeutic results by active targeted CD44 receptor and FR receptor internalization (Lee et al., 2016b). Lai and co-workers recently prepared self-assembled amphiphilic HA-modified adipic dihydrazide (ADH) conjugates loaded with hydrophobic drug curcumin for CD44 targeted delivery. Prepared HA-modified CUR NPs internalized very efficiently into 4T1 and MCF-7 cells which might be due to CD44-mediated endocytosis releasing its cargo intracellularly into lysosomes. Due to HA modification, this nanocarrier showed a significantly higher accumulation into the tumor cells as compared to free CUR and showed prominent tumor growth inhibition *in vivo*. These results were attributed to the EPR effect and CD44-mediated endocytosis (Lai et al., 2021).

#### Metallic Nanoparticles

The various inorganic and organic NPs in the biomedical field have gained significant attention due to their unique structural characteristics and functionality over the past years. Particularly, magnetic iron oxide (Fe3O4) NPs and AuNPs are used in the field of disease diagnosis and treatment therapy. Several properties like magnetic resonance, thermal, X-ray attenuation, and CT imaging makes them very useful in the biomedical field. Due to variability of cancer conditions, it becomes important to design a theranostic platform that would be able to provide both diagnosis and chemotherapy simultaneously. By using these particles, a multifunctional nanoplatform could be possible, that is able to solve complex problems related to cancer *in vitro* and *in vivo* (Li C. et al., 2014; Tian et al., 2014).

Sargazi et al*.* reported HA-conjugated PEGylated MNPs for the targeted delivery of mitoxantrone (MTX). The MNPs were prepared using Fe3O4 and the surface of MNPs were modified by dopamine hydro bromide (DPA -polyethylene glycol to form DPA-PEG-MNPs. DPA was used to prevent NP agglomeration. DPA-PEG-Fe3O4 NPs were conjugated with HA, followed by MTX loading to form MTX-FA-MNPs. *In vitro* cytotoxicity results against MDA-MBA-231 and MCF-7 cell lines revealed that about 32% MDA-MB-231 cell viability was reduced by MTX-FA-MNPs treatment whereas the cytotoxic effect of MTX-FA-MNPs against the MCF-7 cell line was not much higher as that for the MDA-MB-231 cell line. The reason behind this difference was the expression of the CD44 receptor, the MCF-7 cell line expressed a significantly smaller amount of CD44 receptor on its surface as compared to MDA-MB-231 cells. Flow cytometry results of MTX-FA-MNPs exhibited approximately 70.3% apoptosis against MDA-MB-231 cell lines whereas apoptosis against MCF-7 cell lines showed only 5%. These results were attributed to the high affinity of HA-conjugated MNPs towards the CD44 receptor. The significant high affinity of MTX-HA-MNPs compared to HA towards CD44 receptor was also confirmed by molecular docking simulation. Conclusively, HA-mediated MNPs could be a good choice for CD44-targeted delivery of MTX (Sargazi et al., 2018).

Li and co-workers designed a novel multifunctional theranostic approach for diagnosis and photothermal anticancer therapy against different types of cancer. In this study, Fe3O4@Ag NPs were used as the seed to prepare an Fe3O4@Ag nano star (Fe3O4@Ag NSs). The prepared Fe3O4@Ag NSs were used as triple-response NPs such as magnetic resonance (MR), computer tomography (CT), thermal imaging, and photothermal therapy against cancer cells. Fe3O4@Ag NSs were again decorated with polyethyleneimine (PEI) and HA to form Fe3O4@Ag- HA-NSs, in this formulation Fe3O4 was a core of NPs while the star-shaped Au was the shell. The *in vitro* and *in vivo* xenograft tumor model revealed that this multifunctional nanocarrier can be used as a nano probe for effective magnetic resonance and CT imaging. Moreover thermal absorption characteristics allowed it to be used as a nano probe for thermal imaging of a tumor *in vitro* and *in vivo*. HA-mediated cellular uptake of Fe3O4@Au-HA NSs was evaluated in HeLa cells and U87MG cells, both cells were incubated with the same concentration of Au but uptake of Au from Fe3O4@Au-HA NSs in HeLa cells was significantly higher than that of U87MG cells. This difference in cellular uptake was contributed by the expression of the CD44 receptor on the cell lines, HeLa cells were CD44-enriched cells while U87MG cells do not express CD44 receptor. Thus, the role of HA in CD44 receptor-mediated specific cellular internalization of NPs was justified (Li et al., 2015). In another study, Li et al. also evaluated CD44 targeting ability and tumor magnetic resonance imaging of HA-modified MNPs *in vitro* and *in vivo*. The *in vitro* cell cytotoxicity and hemolysis results illustrated that formulation was biocompatible. This study also showed CD44 receptor-dependent therapeutic efficacy *in vitro* and *in vivo* against HeLa and U87MG cells (Li J. et al., 2014).

#### Ceramic Nanoparticles

Ceramic NPs are non-metallic solids, mainly consisting of oxide, carbides, phosphates and carbonates of metals, and metalloids such as calcium, titanium, silicon, etc., generally prepared by heating followed by cooling. These particles have a wide range of use due to a number of favorable properties such as high heat resistance, amorphous, polycrystalline, dense, porous/hollow, and chemical inertness attributes (Sigmund et al., 2006). They can also be used as catalysis, photocatalysis, photodegradation, and imaging agents (Thomas et al., 2015). MSNs could be easily decorated with polymeric moieties on the external surface, which make them more controllable in the field of drug delivery. Recently Yang et al. prepared MSNs for targeted delivery of oxaliplatin (OXL) and miRNA-204-5p. OX-mi-HSMN showed significantly higher uptake efficiency and higher cell cytotoxicity than all other formulations in CD44 overexpressed receptors present on HT-29 cells demonstrating internalization *via* HA receptor-mediated endocytosis, which leads to increased anticancer activity of OX-mi-HSMN. OX-mi-HSMN also showed 43.9% pre-apoptosis which had significantly higher anticancer efficacy in terms of apoptosis potentials. Remarkably, OX-mi-HSMN exhibited significantly higher noticeable downregulation of tumor growth than free OXL and OXL-MSNs. Conclusively, delivery of OXL and miRNA by HA-targeted MSNs showed synergistic anticancer effect against colon cancer (Yang et al., 2019). In another study by Nairi et al., biocompatible MSN-based nanoparticles were prepared. This researcher took three HA samples of different molecular weights, HAS (8–15 kDa), HAM (30–50 kDa), and HAL (90–130 kDa). The formed NPs were denoted as MSN-HAS, MSN-HAM, and MSN-HAL. Zeta potential results showed an increase in negative value with the length of the HA chain. Cellular uptake results for different formulations on HeLa cells at 37°C were investigated by optical and electron microscopy. These results showed that cellular uptake of MSN-NH2/MSN-HAS was higher as compared to MSN-HAM and MSN-HAL, the reason behind the lower internalization of the high molecular weight polymer might be the large size of the sample. A similar study at 4°C exhibited the internalization of MSN-HA samples but not for MSN-NH2, these results suggest that the internalization of samples was mediated *via* two different mechanisms. MSN-NH2 internalization primarily occurred due to electrostatic interactions between cellular phospholipids and MSN-NH2, whereas MSN-HA uptake was mainly driven by CD44 receptor-mediated endocytosis (Nairi et al., 2018). In another study, researchers evaluated the effect of molecular weight of the polymer and methods of preparation on the physicochemical and biological performance of MSNs. In this study, two different MW Has namely HA200 and HA 6.4 kDa were conjugated with MSNs *via* two different methods, in the first method “A,” both MW HAs were preactivated followed by addition of NPs and in the second method “B,” both MW HAs were activated in the presence of NPs; formed NPs were denoted as HA200A, HA6.4A and HA200B, HA6.4B, respectively for methods A and B. All four samples exhibited the same solubility but variation in the loading of HA, texture, surface charge, and stability. Method A could not perform well in these parameters showing low HA loading and stability in biological conditions. In contrast to this, method B performed well with high molecular weight HA, this high MW of HA loading onto the MSNs reflected the biological results of NPs. *In vitro* cellular uptake of different formulations against MDA-MB-231 (CD44^+^) and A2780 (CD44^−^) cell lines showed significantly higher uptake of HA200B, HA6.4B into the MDA-MB-231 (CD44^+^) than A2780 (CD44^−^) cells. More precise uptake of HA6.4B into MDA-MB-231 (CD44^+^) was due to HA-dependent CD44 receptor-mediated endocytosis of formulation (Ricci et al., 2018).

### CD44-Engineered Dendrimer for Cancer Therapy

Between 1970–1990, Buhleier et al. and Tomalia et al. synthesized dendrimers for the first time. Dendrimers have a precise controlled structure, suitable for surface functionalization with different ligands (Kokare et al., 2021). The word dendrimer is derived from a Greek term dendron which means tree, that is because of their distinctive architecture with a number of branching units. Dendrimers may be defined as synthetic polymer macromolecules, with high branching points, 3D globular shape, monodispersity, and a nanometric (1–100 nm) size range. Due to nanoscopic size and monodispersity, the dendrimer is also popularly known as cascade molecules, arborols, dendritic molecules, and nano-metric architectures (Kesharwani et al., 2012, Kesharwani et al., 2014a, Kesharwani et al., 2014b; Kesharwani et al., 2015b; Gawande et al., 2021). The dendrimer architecture is primarily composed of three different domains, i.e., a “core” center consisting of an atom/molecule with at least two identical chemical functional groups, “branches” originating from the core, a repeat unit with at least one branch junction, this repetition is organized in a geometrical progression that results in a series of radially concentric layers known as “generation,” and a “terminal functional group” which determines the properties of the dendritic macromolecules (Fréchet, 1994). The dendrimer is an ideal nanocarrier to achieve passive targeting due to permeability and retention effects in tumor sites. Due to its surface functional group, the dendrimer also offers active targeting of different sites of dendrimers. The use of a plain PAMAM dendrimer was limited to achieve targeted delivery of anticancer molecules due to its intrinsic surface cationic charge, which caused high cytotoxicity because of non-specific interactions with both normal and cancer cells. To solve this problem, there are a number of approach available such as PEGylation, coating with different polymers, and conjugation of targeting ligands. Among these approaches, ligand conjugation is one of the best approaches. Conjugation of ligands with dendrimers not only reduces inherent cytotoxicity of PAMAM but provides some other benefits such as enhancement in solubility, increase in drug loading, sustained/controlled drug release, and improvement in stability, biodistribution, and pharmacokinetics (Duncan and Izzo, 2005; Jain et al., 2010; Kesharwani et al., 2015c; Kesharwani and Iyer, 2015). Kesharwani et al. designed a CD44-targeted 3, 4-difluorobenzylidene curcumin (CDF)-loaded G4 PAMAM dendrimer functionalized with HA and evaluated its therapeutic activity against MiaPaCa-2 and AsPC-1 cells. Initially, HA was conjugated with the NH2 group of dendrimers *via* the EDC coupling reaction. The COOH- group of HA was easily conjugated with the amine functional group of dendrimers by amide bond formation in the presence of EDC, conjugation was confirmed by TEM images, which exhibited 7.6 ± 1.7 and 9.3 ± 1.5 nm sizes for the PAMAM dendrimer and HA-PAMAM dendrimer, respectively. HA-PAMAM-CDF showed 1.42-fold higher entrapment efficiency than PAMAM-CDF, which might be due to HA on the surface of the PAMAM periphery having more space to load a higher amount of CDF. Due to steric hindrance, HA-PAMAM-CDF showed sustained and delayed release of CDF at pH 7.4. HA-PAMAM-CDF comparatively showed better cytotoxicity, due to the CD44 receptor-mediated uptake of the formulation. Moreover, HA-PAMAM-CDF exhibited higher anticancer activity in MiaPaCa-2 cells than AsPC-1 cells because the expression of CD44 receptor was comparatively higher on MiaPaCa-2 cells than AsPC-1 cells. Targeting efficiency of HA-PAMAM-CDF was reconfirmed by the receptor blocked study, here firstly the CD44 receptor was blocked by the presence of an excess of free HA. The IC50 value was found to be 405 ± 6.32 nM for HA-PAMAM-CDF, without a HA-blocked receptor, whereas with the HA-blocked receptor, the formulation exhibited an IC50 value of 694 ± 8.36 nM for HA-PAMAM-CDF. A significant difference was observed in the cytotoxicity curve of HA-PAMAM-CDF as compared to free CDF and PAMAM-CDF, which clearly indicated that the CD44 receptor is a major pathway for HA-PAMAM-CDF uptake. Higher HA-PAMAM-CDF uptake through the CD44 receptor due to the presence of HA on the periphery of the PAMAM was also confirmed by the fluorescence microscopic studies (Kesharwani et al., 2015d). In another similar study, Qi et al. fabricated a dual functional HA-conjugated PAMAM dendrimer for long systemic circulation and active targeting of CD44 receptor and delivery of topotecan HCl (TPT). HA was conjugated with PAMAM in the presence of NHS/EDC to form HA-PAMAM, additionally HA-PAMAM was linked with fluorescence isothiocyanate (FITC) to form FITC-conjugated FA-PAMAM. A hemolytic study revealed that HA conjugation with the PAMAM significantly reduced the hemolytic toxicity of PAMAM at low as well as higher concentrations. HA-PAMAM showed 2.67-fold higher drug encapsulation than PAMAM, due to the presence of HA on the PAMAM surface. Due to HA conjugation, TPT-loaded HA-PAMAM exhibited sustained release of TPT. *In vitro* cytotoxicity study was evaluated in the HCT-116 cell lines, these cell lines are reported to be CD44 + receptor cell lines. After 48 h of incubation, the IC50 value found to be 38.029 nM and 4.966 nM for HA-PAMAM and PAMAM, respectively, clearly indicating that the HA conjugation significantly decreased the cytotoxicity of the PAMAM dendrimer. Cell cytotoxicity for free TPT and TPT-loaded HA-PAMAM were also evaluated in HCT-116 cell lines, the IC50 values were 0.262 and 0.086 nM for the free TPT and TPT-loaded HA-PAMAM, respectively. These results revealed that TPT-loaded HA-PAMAM had relatively higher cytotoxicity at lower concentrations. To evaluate CD44 receptor-mediated uptake of the formulation, the HCT-116 cell line was incubated with FITC-labeled HA-PAMAM with or without HA treatment. Fluorescence microscopy showed competitive binding for the CD44 receptor, meaning a significant reduction in fluorescence intensity of HA-PAMAM-FITC in the pre-incubated HA HCT-116 cell lines. This result was reconfirmed by flow cytometric analysis. Pharmacokinetics of preparation were also evaluated for free TPT, TPT-PAMAM, and TPT-HA-PAMAM in normal rates. MRT was found to be 0.56, 2.53, and 7.09 h for free TPT given in PBS, TPT in PAMAM, and TPT in HA-PAMAM, respectively. TPT-loaded HA-PAMAM exhibited higher MRT as compared to other preparation, as HA may shield all the surface charge of PAMAM and prevent opsonization, thus due to this effect, HA increased the MRT of HA-PAMAM. Tissue distribution results showed 3.6-fold higher TPT distribution in the kidney and 1.7-fold higher distribution in the liver due the HA modification. *In vivo* antitumor results in S-180 mice were also examined for all groups namely, free TPT, PAMAM/TPT, and HA-PAMAM-TPT, here also HA-conjugated PAMAM/TPT was found to have comparatively higher anticancer activity (Qi et al., 2015). Zhanga et al*.* developed mixed dendrimer micelles (MDM) based on dendrimers for the co-delivery of MDM-1-siRNA and DOX, for the inhibition of genes which are involved in the development of MDR. Additionally, a dendriplex was prepared by mixing MDM-1-siRNA with PAMAM *via* electrostatic interactions, HA had no effect on the complexation between PAMAM and siRNA. HA-DOPE/MDM/siRNA and HA/MDM/siRNA dendriplexes were evaluated for stability. Gel electrophoresis results revealed that approximately 85% of siRNA was intact after 30 min. In the presence of RNAse, indicated formulations were able to protect the dendriplexes from RNAse-mediated degradation. Cytotoxicity of free MDM and HA-modified formulations were evaluated in the A2780ADR, MDA-MB-231, and HCT-116 cell lines. Due to the anionic charge of HA, the cationic charge of PAMAM was masked, hence both HA/HA-DOPE functionalizations increased the cytotoxicity of formulations in all cell lines. HA/MDM exhibited comparatively lower cytotoxicity than HA-DOPE/MDM in MDA-MB-231 cell lines, this is because the HA covered the complete surface of MDM. The DOPE moiety promotes strong binding of HA with an MDM structure. Cytotoxicity of combinations, namely MDM loaded with DOX (MDM-D), HA-DOPE-MDM-DOX, and HA-DOPE-MDM-DOX-siMDR-1, was evaluated on all three cell lines; results were DOX concentration and quantity of CD44 receptor expression-dependent. HA-DOPE-MDM-DOX with 10 µM DOX exhibited higher cytotoxicity than MDM-DOX in both HCT116 and MDA-MB-231 cell lines. In A2780 ADR cell lines, HA-mediated cytotoxicity was not observed because of lower CD44 receptor expression in A2780 ADR compared to HCT116 and MDA-MB-231 cell lines. Membrane-bound P-gp was overexpressed in both HCT116 and MDA-MB-231 cell lines; co-delivery of DOX and siRNA in HA-DOPE-MDM can further increase the specificity and cytotoxicity in CD44 and P-gp overexpressed tumor cells (Zhang et al., 2020).

Tang et al*.* designed and developed a synergistic anticancer system to achieve chemotherapy and phototherapy simultaneously. HA-PAMAM-COOH-TMZ (HPCT) and HA-PAMAM-COOH-ICG (HPCI) were prepared which showed spherical, monodispersed, uniform, and better distributed particles than non-modified PAMAM. HPCT exhibited 44.91 and 16.64% EE and LC, respectively for TMZ while HPCI showed 99.87% and 28.55% EE and LC, respectively for ICG. Release of TMZ was significantly increased at pH 5.0, because at this pH hyaluronidase caused enzymatic hydrolysis of HA. For the photothermal therapy, HPCI was better than free ICG because in HPCI, ICG was present in the PAMAM cavity, which made it stable in the solution and in increased laser irradiation. Additionally, HPCI generated higher singlet-oxygen than the free ICG. In cell viability results, HPCT + HPCI + NIR combinedly exhibited the highest anticancer activity and exhibited 17.1% cell viability, which is better than the HPCT + NIR, HPCI + NIR (36.3% cell viability), and ICG + NIR (48.9%) groups. These results established synergistic anticancer activity of HPCT + HPCI under laser irradiation. *In vivo* biological distribution of NPs was studied in nude mice after IV injection in the tail vein that exhibited strong fluorescence in tumor cells 72 h after injection. These results indicated that HPCI and HPCT mainly accumulated in cancer cells which was further proved by NIR irradiation effective 24 h after injection. *In vivo* anticancer results of HPCT + HPCI + NIR groups showed complete disappearance of the tumor. Biodistributions of NPs to the normal tissues were evaluated, showing no damage to normal tissue by treatment of combined therapy. There was no significant change into the level of alanine aminotransferase, aspartate aminotransferase, total bilirubin, creatinine, urea N2, uric acid, and creatine phosphate in tumor-bearing mice and normal mice groups (Tang et al., 2018). Therapeutic benefits of many anticancer molecules were compromised due to MDR, this MDR was mostly due to the Pgp-mediated efflux of drugs. It is considered that nanocarriers escaped from Pgp efflux by endocytosis and internalized drugs into the cytoplasm, but in some cases another protein called major vault protein may be involved in the development of drug resistance, which transport drugs like DOX out from the target site (nucleus) to the cytoplasm (Van Zon et al., 2006; Abbasi et al., 2013). To overcome this problem, Han et al*.* has designed a HA-functionalized PAMAM dendrimer for the codelivery of DOX and MVP-siRNA against MCF-7/ADR cells. Firstly, HA–PAMAM were prepared then DOX was loaded into HA-PAMAM to form DOX-HA-PAMAM. The DOX-HA-PAMAM/siRNA dendriplex was prepared by adding 20 µM of siRNA into the specified amount of DOX-HA-PAMAM solution (1:100 w/w, siRNA: polymer) and incubated for 20 min at RT. Negatively charged siRNA bind electrostatically with positively charged polymers. Gel electrophoresis confirmed the binding ability and stability of siRNA with HA-PAMAM. DOX-HA-PAMAM exhibited higher *in vivo* bioavailability and MRT (AUC, 4.79 h μg/ml and MRT, 21.8 h) compared to the free DOX solution group (AUC, 0.98 h μg/ml and MRT, 3.1 h), which indicated sustained DOX release from HA-coated PAMAM. HA decoration on PAMAM played an important role in these results, due to HA, the nanocarrier was internalized into the tumor cell by overexpressed CD44 receptor-mediated endocytosis. In MCF-7/ADR cells, accumulation of DOX was much less than MCF-7 cells because MCF-7/ADR overexpressed Pgp which caused Pgp-mediated efflux of DOX. DOX loaded into HA-PAMAM avoided/bypassed Pgp and significantly increased the accumulation of DOX into both MCF-7 and MCF-7/ADR cell lines. Pre-treatment with HA or chloropromazine (CPZ) significantly decreased the accumulation of DOX into cell lines, which indicated HA-PAMAM uptake was CD44 receptor-mediated through clathrin-dependent endocytosis. DOX–HA–PAMAM exhibited comparatively higher cytotoxicity (IC50, 11.3 µM) compared to the free DOX solution (IC50, 48.5 µM) in MCF-7/ADR cell lines. Western blot assay revealed that the MVP-siRNA solution and MVP-siRNA lipofectamine 2000 were not effective in downregulating the MVP expression in MCF-7/ADR cells, while PAMAM-HA was able to downregulate MVP protein expression as compared to untreated cells. Intracellular localization of DOX in MCF-7/ADR cells was evaluated by confocal microscopy with or without MVP-siRNA treatment. Initially no significant difference in intracellular DOX fluorescence intensity between free DOX solution and DOX-HA-PAMAM was observed but after 24 h, DOX was observed outside the nucleus, whereas the MVP downregulated cell still exhibited significant strong fluorescent in the nucleus. Conclusive co-delivery of DOX and MVP-siRNA by PAMAM-HA could enter into the nucleus without MVP-siRNA pre-treatment and induce gene silencing and cytotoxicity (Han et al., 2012). A summary of dendrimers used against cancer therapy has been illustrated in Table 3.

**TABLE 3:** Analysis of dendrimers used against tumor cells overexpressing CD44.

Types and generation of dendrimers	Therapeutic agents	Types of cancer	Cell lines	Animal model	References
G4 PAMAM	CDF	Pancreatic cancer	MiaPaCa-2 and AsPC-1 cells	—	Kesharwani et al. (2015c)
G4 PAMAM	Topotecan	Colorectal cancer	HCT-116 cells	Normal rate/S-180 mice	Qi et al. (2015)
G4 PAMAM	DOX and siMDR-1	—	HCT116, A2780 ADR and MDA-MB-231	—	Zhang et al. (2020)
G5 PAMAM	TMZ and ICG	Melanoma	A375 cells	Mice	Tang et al. (2018)
G5 PAMAM	DOX and MVP - siRNA	Breast cancer	MCF-7/ADR and MCF-7	Mice	Han et al. (2012)

### CD44-Engineered Carbon Nanotube for Cancer Therapy

In 1991, Iijima first observed a tubular carbon structure by TEM, called a carbon nanotube (CNT). CNTs can be defined as graphite sheets, wrapped up into cylindrical shapes (Iijima, 1991). It is composed of derivatives of both carbon fibers and fullerene with molecules of 60 carbon atoms arranged in specific tubes. Based on layers, there are two types of carbon nanotubes namely single-walled carbon nanotubes (SWCNTs) and multi-walled carbon nanotubes (MWCNTs). SWCNTs are composed of a single layer of graphene of between 0.4 and 2.0 nm in diameter whereas MWCNTs consist of two or more graphene cylinders with a diameter of 1–3 nm (He et al., 2013). Currently CNTs are under the scrutiny for use as a new tool for biomedical applications. A CNT has many characteristics such as ordered structure with high aspect ratio, low weight, high mechanical strength, high electrical and thermal conductivity, and the length of the CNT is in the form of micrometers with a diameter of about 100 nm (Polizu et al., 2006). Due to these unique characteristics, CNTs have the potential for diverse applications including biomedicals (Lacerda et al., 2006). A CNT can easily cross the plasma membrane and reach directly into the targeted cells because of its needle-shaped structure, and be internalized into the cell by the endocytosis mechanism without causing cell lysis (Cai et al., 2005). The prominent properties, mainly controlled and targeted drug delivery, makes them potential carriers for therapeutic benefit. The multifunctional surface group of a CNT allows them to conjugate different targeting ligands for targeted delivery of many anticancer therapeutic agents. A number of HA-functionalized CNTs were reported for targeting overexpressed CD44 receptor on various tumor cells (Klumpp et al., 2006) (Table 4). Singhai et al. used an MWCNT functionalized with HA and α-Tocopheryl succinate (α-TOS) for the targeted delivery of DOX against TNBC cells. Prepared different formulations namely aminated MWCNTs/DOX, α-TOS-MWCNTs/DOX, HA-MWCNTs/DOX, and α-TOS-HA-MWCNTs/DOX exhibited drug loading of 56.25 ± 1.77, 65.54 ± 2.13, 67.76 ± 2.98, and 76.23 ± 1.76, respectively. α-TOS-HA-MWCNTs/DOX exhibited comparatively higher drug loading compared to other formulations which might be due to conjugation of two different polymers with CNTs, providing a large space to load a large amount of the drug. A cellular uptake study against MDA-MB-231 cell lines revealed that α-TOS-HA-MWCNTs/DOX exhibited significant higher cellular internalization than aminated MWCNTs/DOX, α-TOS-MWCNTs/DOX, HA-MWCNTs/DOX, and free DOX. Plain or free DOX rarely entered into the tumor cell while aminated MWCNTs/DOX exhibited better uptake than plain DOX. Cellular internalization was improved with α-TOS-MWCNTs/DOX but HA-MWCNTs/DOX showed even better cellular internalization than α-TOS-MWCNTs/DOX. Results confirmed the role of HA in cellular uptake by CD44 receptor-mediated endocytosis. *In vitro* cytotoxicity results showed comparatively significant higher cytotoxicity and the lowest IC50 value for α-TOS-HA-MWCNTs/DOX (IC50, 0.810 ± 0.017; *p* < 0 0.001). Combination of both polymers in α-TOS-HA-MWCNTs/DOX (IC50, 0.810 ± 0.017) exhibited lower IC50 than the individual polymer. Dual ligands conjugated on MWCNT surfaces enhanced cellular uptake of α-TOS-HA-MWCNTs/DOX through which more DOX was available to exhibit its therapeutic effect on the tumor cell. This was attributed to CD44 receptor-mediated cell internalization which led to a decrease in IC50 values (Singhai et al., 2020). Prajapati et al. designed gemcitabine-loaded HA-PEG-MWCNTs for colon cancer targeting and evaluated them *in vitro* and *in vivo*. GEM/HA-PEG-MWCNTs exhibited maximum entrapment efficiency 90.2 ± 1.9% which was higher than GEM-MWCNTs, GEM/HA-MWCNTs, and GEM/PEGMWCNTs. An intracellular uptake study of FITC-labeled GEM/HA-MWCNTs exhibited significant higher cellular uptake among all other formulations, this was attributed to uptake of the formulation through HA receptor-mediated endocytosis. GEM/HA-PEG-MWCNTs showed lower cellular uptake than GEM/HA-MWCNTs, which might be due to the hydrophilic chain of PEG on the CNT inhibiting interactions between the formulation and cell membrane. *In vitro* cytotoxicity against HT-29 cells showed that HA-coated CNTs exhibited comparatively higher cytotoxicity than non-HA-coated CNTs. GEM/HA-PEG-MWCNTs were relatively more compatible in the blood (less hemolytic) than other preparations. Pharmacokinetic parameters of GEM such as C_max_, t_1/2_, MRT, and AUC were comparatively improved by GEM/HA-PEG-MWCNTs. *In vivo* antitumor activity was investigated in tumor-bearing female rats (Sprague-Dawley strain) by measuring tumor growth. GEM/HA-MWCNTs and GEM/HAPEG-MWCNTs both decreased tumor volume significantly compared to free GEM. GEM/HA-MWCNTs exhibited higher antitumor activity than GEM/HAPEG-MWCNTs because of receptor-mediated accumulation and intracellular internalization (Prajapati et al., 2019). Leu et al. prepared a targeted delivery system for DOX against TNBC cell lines. They used HA-conjugated aminated single-walled carbon nanotubes (NH2 SWCNTs), where HA was non-covalently coated on to the CNT surface. Surface morphology and size of CNTs was significantly changed after HA coating, the size of nano-NH2-SWCNTs and SWCNTs-DOX was around 2000 nm which was reduced to around 262.5 ± 0.9 nm after HA coating. As reported in many studies, adsorption and desorption are the main characteristics of CNTs. Hence in this study, SWCNTs-DOX-HA showed 81.5 ± 1.0% DOX adsorption. *In vitro* drug release and desorption of DOX from SWCNTs-DOX-HA was pH-triggered. At pH 7.4, desorption of DOX from SWCNTs-DOX and SWCNTs-DOX-HA was slow which was around 11% and 8% in 24 h, respectively, whereas in pH 5.5, the PBS desorption rate of DOX from SWCNTs-DOX-HA increased to 34.5% in 24 h. *In vitro* cellular uptake of SWCNTs-DOX and SWCNTs-DOX-HA into the MDA-MB-231 cell lines were also evaluated, flow cytometry results revealed that SWCNTs-DOX-HA had 2.1-fold higher uptake of DOX than the SWCNTs-DOX. Cellular uptake results were reconfirmed by confocal microscopy, which was also consistent with cellular uptake results of flow cytometry. Higher cellular uptake of SWCNTs-DOX-HA than SWCNTs-DOX was possibly due to HA receptor-mediated endocytosis. The SRB assay revealed that SWCNTs-DOX-HA and SWCNTs-DOX showed dose-dependent anticancer activity, additionally SWCNTs-DOX-HA exhibited significant higher anticancer activity as well as MDA-MB-231 cell migration inhibition than SWCNTs-DOX, which was maximum at 1.0 μg/ml. The *in vitro* apoptosis rate by flow cytometry was 2.33 ± 0.56%, 9.25 ± 1.62%, 37.72 ± 1.03%, and 73.45 ± 1.54% respectively for control, SWCNTs-DOX, SWCNTs-DOX-HA, and free DOX groups. SWCNTs-DOX-HA was better than SWCNTs-DOX, the difference in the results was mainly attributed to HA modification (Liu Z. et al., 2019). Cao and co-worker also evaluated the targeted ability of DOX-loaded PEI-modified MWCNTs covalently attached with FI and FA, the final prepared product was denoted as MWCNT/PEI-FI-HA/DOX. As mentioned earlier, loading and unloading of drugs onto the CNTs was pH-dependent, here the MWCNT/PEI-FI-HA/DOX complex showed DOX loading was 72.0% at basic pH which was significantly higher than at an acidic pH. In an acidic condition, DOX was protonated showing increased hydrophilicity which prohibited hydrophobic π-π interactions with CNTs, while at basic pH DOX was deprotonated and quite hydrophobic, which facilitated hydrophobic π-π interaction with CNTs leading to higher drug loading. Due to effect of the pH on loading/unloading, the DOX release rate was higher at acidic pH (5.8) at 54.6% whereas at physiological pH it was 40.8% after 48 h pH-triggered release of DOX from prepared complexes was beneficial for tumor targeting. The CD44 receptor targeted efficiency of HA-conjugated complex was investigated in HeLa cells and normal L929 cells. Flow cytometry results of prepared complexes showed seven times higher uptake into HeLa cells than L929 cells. Obtained results confirmed the idea of CD44 receptor-mediated endocytosis of prepared complexes into the HeLa cells, enhancing its uptake. Confocal microscopy results were also consistent with flow cytometry results. An MTT assay was also performed to determine % viability in both HeLa and L929 cells treated with prepared complexes. After 2 h of incubation, HeLa cells showed 50% viability while almost all L929 cells were alive. All these results indicated HA mediated targeted inhibition of tumor cells *via* CD44-mediated endocytosis and intracellular uptake (Cao et al., 2015). Faraj et al. reported a novel approach of combination therapy against breast cancer cells and cancer stem cells (CSCs). In this study, they loaded paclitaxel and salinomycin (SAL) onto SWCNTs, and biocompatible CD44 antibody was conjugated on SWCNT surfaces by a hydrazone linker. Here, prepared conjugates were denoted as SWCNT-PEG-Hyd-5HP-SAL-CD44 and SWCNT-PEG-Hyd-LEV-PTX-CD44 for the salinomycin and PTX-loaded CNTs, respectively. The *in vitro* cytotoxicity study was evaluated using CSC−, CSC^+^, and MDA-MB-231 cell lines. These cell lines were incubated with different formulation samples for 24, 48, and 72 h. The MTT assay revealed that the SAL and PTX-loaded SWCNT-treated cells exhibited a prominent effect on cell viability compared to free drugs, moreover, co-delivery of both formulations showed maximum cytotoxic activity as compared to individual delivery. Combined treatment with SWCNT-SAL and SWCNT-PTX showed cell viability of 20.3 ± 1.2%, 26.1 ± 1.3%, and 22.9 ± 0.9% respectively against MDA-MB-231, CSC^−^, and CSC^+^ after 72 h of incubation. To confirm these results, bioluminescence signal (BLI) measurement was performed on all three cell lines. Combined delivery of both drugs by nanocarriers showed a significant reduction in bioluminescence. Like the above results, BLI also confirmed a higher antitumor capability of SWCNT-SAL and SWCNT-PTX than free drugs. In this study, *in vivo* anticancer activity of the drug-loaded SWCNT conjugate was also evaluated by MRI and BLI imaging. The tumor volume of MDA-MB-231 cell-bearing mice was 200 ± 20 mm^3^ observed by non-invasive MRI and radiance efficiency of 47.5 ± 2.1 × 10^6^ ρ/s/cm^2^/sr by non-invasive BLI. The radiance efficiency in the control mice group (non-treated) gradually increased with time, which reached up to 139.6 ± 8.4 × 10^6^ in 5 weeks. The signal of BLI was reduced after the 3rd dose of salinomycin injection, it was reduced in a significantly higher amount after paclitaxel injection and injection of combined drugs. Radiance efficiency reduction reached 75.8 ± 7.5 × 10^6^ in the SAL + PTX-injected mice group up to the last investigation. An equivalent dose of both drugs reached a radiance efficiency of 35.6 ± 7.1 × 10^6^ (salinomycin formulations) and 23.7 ± 8.9 × 10^6^ (paclitaxel formulations) at 5 weeks of investigation. Combined drug conjugates significantly reduced the radiance efficiency to 2.4 ± 2.1 × 10^6^ ρ/s/cm^2^/sr. MRI results were also consistent with BRI results; the tumor volume of the non-treated group was 615 ± 50 mm^3^ at the last MRI measurement, whereas the combined drug-conjugated treatment group exhibited a tumor volume of 15.5 ± 13.0 mm^3^ up to the last measurement which was significant less than all other formulations. Here CD44 antibody-conjugated SWCNTs had a very significant role in selective active targeting of CSCs, which improved the therapeutic efficiency of this tumor therapy (Al Faraj et al., 2016). Another study reported a CD44 targeting novel nanotube which is known as a halloysite nanotube and modified with HA for acquiring targeted therapy. As evidenced by confocal microscopy, the cellular uptake was enhanced due to the tumor homing effect. Moreover, the preparation showed improved therapeutic effect due to internalization of DOX inside the cell (Mo et al., 2021).

**TABLE 4:** Summary of CNT-targeted CD44 cells.

Types of CNTs	Therapeutic agents	Types of cancer	Types of study (*in vitro*/*in vivo*)	Cell lines	Animal model	References
MWCNTs	DOX	Breast Cancer	*In vitro*	MDA-MB-231	—	Singhai et al. (2020)
MWCNTs	GEM	Colon cancer	Both	HT-29	Sprague-Dawley rat	Prajapati et al. (2019)
SWCNTs	DOX	TNBC	*In vitro*	MDA-MB-231	—	Liu Z et al. (2019)
MWCNTs	DOX	Cervical cancer	*In vitro*	HeLa and L929	—	Cao et al. (2015)
SWCNTS	SAL and PTX	Breast cancer	Both	MDA-MB-231 and CSC-/CSC ^+^	Mice	Al Faraj et al. (2016)

### CD44-Engineered Nanoemulsion for Cancer Therapy

The term “nanoemulsion” (NE) or mini-emulsion means fine oil/water or water/oil dispersion stabilized by an interfacial film of a surfactant molecule with a droplet size range of 20–600 nm (Jaiswal et al., 2015). Due to their small size, nanoemulsions are transparent in appearance. Many other unique properties of NEs include amorphous surface, solid sphere, high drug loading, and negatively charged particles. Nanoemulsions are mainly composed of oil, surfactant, and co-surfactant. They are extensively used as a carrier for poorly soluble chemotherapeutic drugs to enhance their solubility *via* their solubilizing capacity (Tadros et al., 2004). As a parenteral drug carrier, NEs have numerous advantages over other nanocarriers such as simple manufacturing process, high BA and solubilizing capacity, and long shelf-life, etc. Attachment of targeting moieties to NEs is quite challenging because of the weak bond with targeting moieties. Therefore, a highly advanced approach and thorough study is needed before preparing a tumor-targeted drug delivery system (Gupta et al., 2015; Hörmann and Zimmer, 2016). Kim et al*.* used hyaluronan-modified nanoemulsions (HNEs) for the targeted delivery of poorly soluble drug paclitaxel. The stability results revealed that HNEs were stable for 96 h which was significantly higher than Taxol^®^, which was stable only for 72 h. HNEs showed significantly higher uptake into the SK-OV-3 compared to the OVCAR cell lines, uptake efficiency of HNEs into the SK-OV-3 cell lines was directly related to the quantity of HA attached to the HNE surface. SK-OV-3 and OVCAR-3 cell lines displayed around 10 times higher targeting affinity for HNEs than the NEs. These results were further confirmed by confocal microscopy; confocal images showed that in SK-OV-3 cells, a remarkably higher binding affinity and cellular uptake was observed. Finally, researchers concluded that HA-modified NEs were stable, were able to solubilize poorly soluble drugs, were highly stable, and had high drug loading with efficient active targeting ability (Kim and Park, 2017a). In another study by the same research group, PTX-loaded HA-modified solid NEs (PTX-HSNs) were evaluated. In this study, the nanoemulsion was prepared by the high pressure homogenization method, the concentration of PTX loading was reduced to 3 mg/ml with approximately 100% entrapment efficiency. The HA-modified PTX-NEs exhibited 100-fold *in vitro* binding affinity in SK-OV-3 and OVCAR-3 cells as compared to non-HA-modified PTX nanoemulsions. The targeted preparation showed efficient tumor reduction with enhanced pharmacokinetic profile (Kim and Park, 2017b). Wang et al. developed a CD44-targeted microemulsion loaded with shikonin (SKN) and docetaxel (DTX) for the treatment of glioma. Targeted delivery of chemotherapeutic agents to the brain is a very challenging task due to the blood-brain barrier (BBB). The SKN and DTX-loaded microemulsion was bifunctionalized with A1411 aptamer and HA that exhibited targeted delivery *via* specifically binding with CD44/nucleolin-overexpressed glioma. The *in vitro* cytotoxicity study against U87 cells treated with HA loaded with both SKN and DTX showed an IC50 value of 3.4 ± 0.3 mg/ml which was reduced to 2.0 ± 0.3 mg/ml when U87 cells were treated with the HA-modified microemulsion, the IC50 value further declined after the conjugation of aptamer AS1411. The dual targeted preparation enhanced the apoptosis of U87 cells by 71.3 ± 4.2%. Cancer stem cell-enriched U87 cell spheroids were efficiently inhibited by the bifunctional system leading to downregulation of CD133 expression.

The apparent permeability coefficient of the prepared microemulsion was increased as assessed by an artificial BBB model. Additionally, targeted formulations significantly inhibited the growth of brain tumors and showed higher survival period than non-targeted groups. Conclusively, the HA/A1411-modified microemulsion exhibited its superior activity due to effective targeted delivery of the formulation at the targeted site and receptor-mediated endocytosis (Wang et al., 2019). The abovementioned studies proved that emulsions can be used as nanocarriers for the CD44-mediated targeted delivery of many chemotherapeutics (Table 5).

**TABLE 5:** Examples of nanoemulsion-targeted CD44 cells.

Oils, surfactants, and other materials	Therapeutic agents	Types of study (*in vitro*/*in vivo*)	Cancer types	Cell lines	Animal model	References
Soyabean oil, polysorbate 80, dl-a-tocopheryl acetate and Fecl3	PTX	*In vitro*	Ovarian	SK-OV-3 and OVCAR-3	—	Kim and Park, (2017a)
Soyabean oil, polysorbate 80, dl-a-tocopheryl acetate, and Fecl3	PTX	Both	Ovarian	SK-OV-3 and OVCAR-3	BALB/cAnNCrjBgi-nu/nu nude mice	Kim and Park, (2017b)
Mal-DSPE-PEG	Shikonin and DTX	Both	Brain	U87 cell	BALB/C (nu/nu) nude mice	Wang et al. (2019)

### CD44-Engineered Micelles for Cancer Therapy

Micelles are lipidic nanocarriers that assemble themselves in a spherical form in aqueous solutions. They have an amphiphilic architecture which contains both a hydrophobic region (a long hydrophobic chain “core”) and hydrophilic regions (a polar head group “shell”) (Oh et al., 2004). The hydrophobic core of micelles enables them to load hydrophobic drugs while the hydrophilic shell allows binding of water-soluble drugs (Adams et al., 2003). Micelles are biocompatible, stable at biological conditions, and can be used to solubilize highly insoluble pharmaceutical agents/drugs. These properties led to development of many drug-loaded micelles, some of which are in the preclinical and clinical stages currently (Sahoo and Labhasetwar, 2003; Kateb et al., 2011). Micelles possess important characteristics such as nanometric size (diameter <50 nm) and hydrophilicity providing protection for polymeric micelles from elimination by the RES, leading to enhanced retention time and direct delivery to tumor cells. Targeted delivery of anticancer molecules by micelles may be achieved by the ERP effect, or employing several block copolymers, or by conjugating specific targeting ligands to the special surface functional groups of micelles (Dabholkar et al., 2006). Micelles can be easily conjugated with hyaluronic acid (HA) for active tumor targeting, and can efficiently deliver drugs intracellularly by binding with its specific CD44 receptor which has been illustrated in Table 6. It was reported in several studies that CD44 receptors overexpressed in several cancer cells causes MDR. Kesharwani et al. has designed a HA-linked co-poly (styrene maleic acid) (HA-SMA) nano-micellar system for targeted delivery of anticancer molecules (CDF) for the treatment of MDR pancreatic cancer. Nano-micelles (HA-SMA-CDF) were characterized by DLS, and TEMDLS exhibited 96.5 ± 3.1 nm particle size and 0.228 ± 0.14 PDI for SMA-CDF, while 114.1 ± 2.7 nm particle size and 0.236 ± 0.11 PDI was obtained for the HA-SMA-CDF. TEM images showed a particle size range of 70.3 ± 1.8 and 89.5 ± 2.8 nm respectively for SMA-CDF and HA-SMA-CDF with smooth surface and spherical geometry. HA conjugation significantly improved the entrapment efficiency of CDF due to HA that enhanced the CDF interaction with the nano-micelles. HA-SMA-CDF was found to be sufficiently stable at RT in the dark than at 0°C/60°C. The percentage of cell viability was found to be 28.47 ± 2.29%, 15.62 ± 5.74%, and 8.51 ± 1.99% for CDF, CDF-SMA, and HA-SMA-CDF, respectively at a 1,000 nM equivalent concentration in MiaPaCa-2 cells. Similar results were observed in AsPC-1 cells, where HA conjugates showed significantly higher anticancer activity. HA-SMA-CDF also exhibited comparatively lower IC50 values in both MiaPaCa- 2 and AsPC-1 cell lines. To confirm CD44 receptor-mediated cellular internalization, a receptor blocked study was performed. For HA-SMA-CDF, the IC50 value obtained was 182 ± 6.68 nM before blocking by excess HA, whereas the value changed to 234 ± 8.24 nM after pre-treatment of cells with HA. These results clearly established that pre-treated cell lines with free HA significantly enhanced the IC50 values which means pre-treated free HA molecules occupied CD44 receptors and availability of the CD44 receptor for HA-SMA-CDF binding was limited and competitive. Western blot results revealed a significant reduction in CD44 expression level in HA-SMA-CDF-treated cells compared to free CDF and SMA-CDF-treated cells (Kesharwani et al., 2015b). Similarly, Wang et al*.* has designed CDF-loaded HA-SMA-TPGS nano-micelles for CD44-targeted delivery, specially functionalized for NIR imaging. Targeted (HA-SMA-TPGS-CDF) preparation exhibited strong red fluorescence intensity compared to the non-targeted preparation that indicates targeted formulations accumulated faster than non-targeted preparations. This higher accumulation of HA-SMA-TPGS-CDF is possibly due to the high affinity of the HA ligand towards the CD44 receptor. Anticancer activity was also found to be highest for HA-SMA-TPGS-CDF as compared to free CDF and SMA-TPGS-CDF against both cell lines. The high cell killing activity of HA-SMA-TPGS-CDF was possibly due to active tumor targeting due to CD44 receptor-mediated endocytosis. The targeted formulation induced significantly higher apoptosis than free CDF and non-targeted formulations. In this study, researchers have also described the mechanistic pathway behind the apoptosis of TNBC cells, as apoptosis was induced due to the downregulation of NF-κB and upregulation of PTEN in the TNBC cell lines (Kesharwani et al., 2015b). A western blot study displayed significant upregulation of PTEN and downregulation of PTEN and NF-κB protein expression in the HA-SMA-TPGS-CDF-treated cells as compared to free CDF and SMA-TPGS-CDF, and HA-targeted ability of HA-SMA-TPGS was evaluated *in vivo* for the early diagnosis of TNBC by NIR imaging. HA-SMA-TPGS or SMA-TPGS was conjugated with S0456 high contrast dye, which revealed significantly higher fluorescence intensity in the tumor tissue than other normal tissues also indicating the tumor homing effect by the ligand (Wang et al., 2018). Recently, Yang and co-workers developed stable hybrid micelles for targeted delivery of chemotherapeutic agents DOX against TNBC. Here stable hybrid micelles (HT-LT) were prepared by mixing hyaluronic acid-D-α-tocopheryl succinate (HA-TOS, HT) and low molecular weight heparin-TOS (LT). Prepared HT-LT NPs had better stability, prolonged release at pH 5.5 which might be due to breaking of the ester bond. The western blot technique was utilized to investigate the expression of CD44 receptor on different cell lines. It was estimated that CD44 was expressed on 4T1, CT26, and MDA-MB-231 cells but much less in NIH 3T3 cells. CLSM results showed a stronger uptake signal for HT-LT than LT and HT in 4T1 cells. Lysosomal release was also evaluated by lysotracker, which exhibited a strong signal for HT-LT into the lysosome which was due to breaking of the ester bond due to presence of esterases that enabled prolonged release of DOX. Here HT-LT showed 2.2 times higher (highest) uptake efficiency of DOX in 4T1 cells whereas LT showed 1.5 times uptake than that with the lowest efficiency. *In vitro* cellular migration was effectively inhibited by HT-LT NPs more than other formulations. *In vivo* tumor targeting was investigated by loading HT, LT, and HT-LT NPs with fluorescence probe DiD in place of DOX and observed under CLSM. DiD-loaded HT-LT NPs exhibited comparatively stronger fluorescence in the tumor site that commenced after 1 h of administration and persisted for 24 h. Additionally, *ex vivo* tumor images also showed increased tumor accumulation and decreased liver distribution of HT-LT/DiD NPs than others. *In vivo* antitumor activity was investigated in 4T1-bearing Balb/c female mice, the tumor growth inhibition rate was calculated as 20.96%, 32.60%, 46.89%, and 77.39%, respectively for free DOX, HT/DOX NPs, LT/DOX NPs, and HT-LT/DOX NPs. Ki -67 is a marker for cell growth, here researchers found high expression of Ki -67 in the PBS group whereas lower expression in the HT-LT/DOX NP group. These results confirmed the superiority of HT-LT/DOX NP over other formulations (Yang et al., 2020). In another study, Du et al. has developed biocompatible, pH-sensitive, and charge-conversion micelles bearing a half-generation PAMAM dendrimer (G4.5 sPA) decorated with HA-conjugated poly (lactide) (PLA) and loaded with docetaxel. Formed micelles were uniformly distributed, spherical in shape, and had a smooth surface. As reported previously, HA could improve biocompatibility of polymers of carriers with drugs. Here the cationic surface charge of PALA was masked by HA to improve the biocompatibility of micelles. Drug release results revealed that the drug release from the formulation was dependent on HA degradation, which was HAase-mediated and pH-dependent, hence HA-conjugated micelles reached the tumor site through CD44 receptor-mediated tumor targeting, HA was degraded in the tumor site due to the effect of pH and HAase. Formulations such as free docetaxel (DTX), Taxotere^®^, PALA-DTX, and HA-PALA-DTX were compared for their cytotoxicity against MCF-7 cell lines, in this, HA-PALA-DTX exhibited significantly higher cytotoxicity than free DTX and Taxotere^®^. The IC50 value for HA-PALA-DTX was found to be 5.92 ng/ml which was notably lower than that of free DTX (264.91 ng/ml) and Taxotere^®^ (76.56 ng/ml). Cellular uptake of different coumarin-6 (C6)-conjugated formulations were analyzed by CLSM, C6-HA-PALA showed significantly higher intracellular fluorescence intensity than other preparations in the MCF-7 cell lines. *In vivo* biodistribution results by *in vivo* imaging and results of DiR-loaded micelles groups (DiR-HA-PALA) showed the majority of fluorescence at the tumor site and this fluorescence persisted for 12 h, whereas free DiR was not found at the tumor site. The HA modification formulation was internalized by CD44-mediated endocytosis. *In vivo* antitumor activity in the S180 tumor-bearing mice HA-PALA-DTX-treated group showed approximately 72.32% tumor cell inhibition which was highest amongst all other groups. This highest anticancer activity was attributed to the HA modification of micelles. HA-modified formulations also performed well in preliminary *in vivo* safety evaluation. Hence the prepared carrier was viable and efficient to use for targeted delivery of chemotherapeutic agents (Du et al., 2018). Zhong et al. designed and developed similar types of preparation consisting of paclitaxel-loaded HA-modified -b- dendritic oligoglycerol (HA-dOG-PTX) polymeric micelles (PM). Prepared PM exhibited a diameter of 155 nm, low CMC (6.8 mg/L), and 0.12 PDI. Release of PTX from the HA-modified formulation was pH-dependent which showed 20%, 48%, and 81% release at pH- 7.4, pH 6.0, and pH 5.0, respectively. At acidic pH, maximum released PTX was obtained which might be due to degradation of HA at low pH. *In vivo* pharmacokinetic parameters evaluated in the distribution phase at t_1/2_ were equal to 1.63 and 0.12 h, whereas the elimination phase half-lives were 4.32 and 0.23 h, respectively for HA-dOG-PTX-PM and Taxol. HA-dOG-PTX-PM had an AUC of 158.4 mg x h/L, that was 68 times higher than free PTX (2.3 mg x h/L). *In vivo* tumor targeting activity was similar to the above study, where DiR-HA-dOG-PTX-PM exhibited significantly higher fluorescence activity 4 h after injection that reached maximum after 12 h and persisted for 48 h post injection. This indicates that DiR-HA-dOG-PTX-PM was up taken very efficiently by MCF-7 cell lines. Thus, HA modification improved the pharmacokinetics, biodistribution, and *in vivo* antitumor activity due to CD44 receptor targeting (Zhong et al., 2016). In another study, Liu et al. designed p -sensitive dual-functionalized HA–deoxycholic acid histidine (HA-DOCA-His) micelles for CD44-targeted delivery of PTX and to overcome MDR. PTX-loaded HA-DOCA-His showed significantly improved cytotoxicity having greater MDR overcoming activity against MCF-7/ADR cell lines, this was due to the maximum intracellular accumulation of PTX *via* the CD44 receptor-mediated pathway and endosomal pH-triggered release of PTX. Pharmacokinetic parameters such as long blood circulation time, larger AUC, and less V_d_ and CL were achieved by PTX-loaded HA-DOCA-His which was comparatively better than PTX. PTX-loaded HA-DOCA-His exhibited significantly higher antitumor activity in MCF-7/Adr tumor-bearing mice compared to PTX/HA-DOCA micelles and free PTX. Conclusively, PTX-loaded HA-DOCA-His showed better antitumor activity as well as established outstanding MDR reversal ability (Liu et al., 2018). Similarly, Soleymani et al. has designed mixed nano-micelles for targeted delivery of curcumin. In this study, hydrophobic curcumin was encapsulated into the hydrophobic cavity of pluronic F127/dodecyl dimethyl ammonium bromide (PD) *via* the film hydration method, after curcumin encapsulation, the positively charged surface of micelles was interacted with negatively charged HA through electrostatic interaction. HA-coated micelles showed a change in average hydrodynamic size from 19.8 to 35.8 nm and also increased *in vitro* cytotoxicity against MDA-MB-231 cell lines compared to non-coated micelles and free micelles. The IC50 value for HA-coated PD was found to be 2.83 μg/ml which was less than free curcumin and non-HA coated micelles. On the basis of these results, the author suggested that curcumin-loaded HA-PD micelles would be a potential system for targeted delivery of hydrophobic drugs to breast cancer as well as other cancers in which the CD44 receptor was overexpressed (Soleymani et al., 2021). Finally, we can conclude that the variety of micelles modified by HA are able to deliver drug molecules to overexpressed CD44 receptors on various cancer cells.

**TABLE 6:** Examples of micelle-targeted CD44 cells.

Main characteristics of micelles	Therapeutic agents	Types of study (*in vitro*/*in vivo*)	Types of cancer	Cell lines	Animal model	References
Nano micelles	CDF	*In vitro*	Pancreatic	MiaPaCa-2 and AsPC-1	—	Kesharwani et al. (2015b)
Nano micelles	CDF	Both	TNBC	MDAMB- 231 and MDAMB- 268	Mice	Wang et al. (2018)
Hybrid micelles	Dox	Both	TNBC	4T1, CT26, MDA-MB-231, and NIH 3T3	Female mice	Yang et al. (2020)
pH-sensitive micelles	DTX	Both	Breast cancer	MCF-7	Mice	Du et al. (2018)
pH-sensitive micelles	PTX	Both	Breast cancer	MCF-7	Mice	Zhong et al. (2016)
pH-sensitive micelles	PTX	Both	Breast cancer	MCF-7	Mice	Liu et al. (2018)
Mixed micelles	Curcumin	*In vitro*	TNBC	MDA-MB-231	—	Soleymani et al. (2021)

### CD44-Engineered Quantum Dots for Cancer Therapy

**FIGURE 6:**
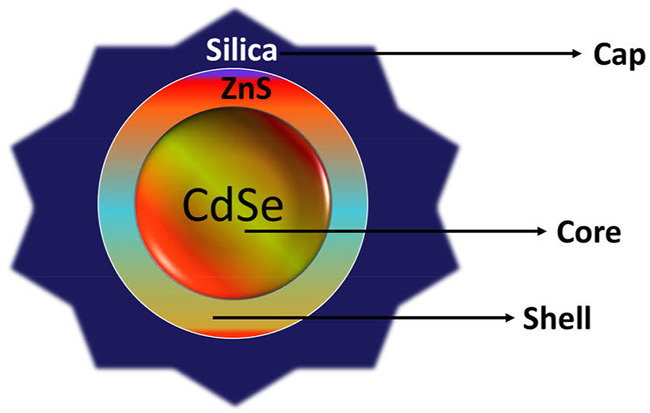
Schematic illustration of quantum dots.

In 1980, Russian physicist Alexei Ekimov first discovered quantum dots (QDs) in solids (glass crystals), later in 1982, a chemist from the US, Louis E. Brus discovered QDs in colloidal solutions (Tokihiro and Hanamura, 1984; Zhu et al., 2013). In 1990, Canham reported optical properties of QDs for first time in silicon (Canham, 1990). QDs are most commonly used for drug delivery and targeting as well as diagnosis and imaging purposes, along with this, QDs are also used for sensing DNA and oligonucleotides (Jaiswal et al., 2004; Mitchell et al., 1999). Quantum dots are semiconductor nanosized crystals having optical and electronic properties which are different from the larger particles because of quantum mechanics. Generally, QDs are mainly composed of three parts, i.e., first, the “core” made up of semiconductor materials, second, the “shell,” a coating that surrounds the semiconductor core, which improves the optical feature of QDs, and third, the “cap” outer layer of QDs encapsulating both the core and shell, mainly consisting of materials like silica, that improve solubility (Figure 6) (Aqel et al., 2012; Chen et al., 2006). There are four basic steps of QD preparation such as core synthesis, shell growth, aqueous solubility, and biomolecular attachment which are involved in the design of QDs for biological applications (Mishra et al., 2017). Steps of QDs like shell growth and surface functionalization enhance stability and photoluminescence of the QD core. QDs are gaining the significant attention of researchers as a part of the technological future, demonstrating various unique physicochemical properties which are required for imaging and targeting purposes into the living cell and whole animals. There are many unique features of QDs like optical properties, aqueous solubility, lower cytotoxicity, biocompatibility, and reduced cost which make them preferred nanocarriers for drug delivery and targeting delivery in the cancer patient (Gerion, 2007; Liu et al., 2012). Recently, Karakoçak et al. used HA-modified nitrogen carbon quantum dots (CQDs) for bioimaging of tumor cells. Firstly, HA-modified nitrogen CQDs were prepared by the coupling reaction of the NH2 group of nCQDs and the COOH group of HA. An *in vitro* comparative biocompatibility study evaluated the dots in both ARPE-19 and CHO cells lines, and revealed that the HA-modified nitrogen CQDs exhibited enhanced biocompatibility in both cell lines and significantly decreased the intracellular ROS concentration in both cell lines. HA-mediated cellular internalization was confirmed by *in vitro* investigation of HA-modified nitrogen CQDs and nitrogen CQDs treated with both ARPE-19 and CHO cells lines, CLSM imaging of these cell lines revealed that HA-modified nitrogen CQDs were efficiently internalized into both cell lines. When the same study was performed on additional cells pre-treated with HA, which remarkably resulted in cellular internalization of HA-modified nitrogen CQDs into both cells, there was not much change in internalizations of nitrogen CQDs. The selective cellular uptake of HA-modified nitrogen CQD and nCQD conjugates was reconfirmed by treating NIH 3T3, ARPE-19, and CHO cell lines with HA-modified nitrogen CQDs. *In vitro* results showed that uptake of HA-modified nitrogen CQDs into the NIH 3T3 cell was comparatively less than that of ARPE-19 cells and CHO cells, this difference in uptake of HA-modified nitrogen CQDs was related to the expression of CD44 receptor in the cell lines. The NIH 3T3 cell lines had much lower expression of CD44 receptor than ARPE-19 cells and CHO cells, so that NIH3T3 cells exhibited less cellular uptake. These results suggested that the HA-modified nitrogen CQDs were facilitated *via* CD44 receptor-mediated internalization by selective HA and CD44 binding. *In vivo* results also revealed that HA-modified nitrogen CQDs accumulated in the tumor site 15 min after injection and the fluorescence signal for HA-modified nitrogen CQDs was detected at the tumor site whereas no fluorescence signal was found for non-HA-conjugated nCQDs. A similar pattern of fluorescence signal was observed in another experiment, in which HA-modified nitrogen CQDs and non-HA-conjugated nCQDs were given *via* IV to mice bearing WHIM4 tumors, 150 min after injection a fluorescence signal at the tumor site was observed for HA-modified nitrogen CQDs, but there was no signal for non-HA-conjugated nCQDs at the tumor site. These results further confirmed the ability of HA-modified nitrogen CQDs to target CD44 receptor (Karakoçak et al., 2021). In another study, Zhang et al. reported HA-functionalized CQDs, prepared by the hydrothermal carbonization method. Formed HA-CQDs were stable, biocompatible, highly hydrophilic, and had uniform size distribution with a heterogeneous multi-layered solid structure. A CCK8 assay showed that the prepared HA-CQDs were biocompatible with Hela and MCF-7 cells. Due to excellent fluorescence properties of HA-functionalized CQDs, labelled MCF-7 and Hela cells could be examined and noticed by using CLSM and flow cytometry. In HA-CQD-treated HeLa and U251 cells, CLSM images exhibited that the HA-CQDs were localized to the cytoplasmic regions, mainly around the nucleus, it seemed that a major amount of HA-CQDs was internalized by tumor cells which could be controlled by receptor-mediated endocytosis. CD44 receptor-mediated cellular internalization was also confirmed by the quantitative analysis *via* flow cytometry, these results revealed that the HeLa cells efficiently took up HA-CQDs while MCF-7 took up a very small amount of HA-CQDs. Notably, internalization of HA-CQDs in HeLa cells decreased when HeLa cells were pre-treated with free HA, but there was not much change in uptake of HA-CQDs in the MCF-7 cells. This is due to the expression of CD44 receptor on HeLa cells which was significantly higher than that in MCF-7 cell lines, so more CD44 receptors were able to selectively bind with HA and get internalized very efficiently. These outcomes were attributed to HA receptor-mediated endocytosis (Zhang et al., 2016). Wang et al. synthesized a HA-modified cysteamine polymer, encapsulating (CdSe) CdZnS QDs to form a final HA polymer-wrapped QD *via* the reverse micelle method. The HA polymer-wrapped QD showed enough stability in PBS and BSA mixed culture medium and in the entire pH range. To assess biocompatibility, MDA-MB-231 cell lines were treated with the HA polymer-wrapped QD, and 83% viability was found even 24 h after incubation. This result revealed that the HA polymer-wrapped QD was biocompatible. CD44-targeted imaging was investigated in MCF-7 and MD-MB-231 cell lines, the NIH/3T3 cell line was used as negative control. All these cell lines were treated with different concentrations of HA polymer-wrapped QDs, and strong fluorescence was observed on MCF-7 and MD-MB-231 cell lines but not on NIH/3T3 (CD44 ^-^) cell lines. These results showed the targeted ability of the HA polymer-wrapped QDs. The receptor blocked assay also confirmed the CD44 receptor imaging of HA polymer-wrapped QD-treated MCF-7 cell lines (Wang et al., 2014). In another recent study, similar results were obtained for multifunctional redox-sensitive HA-modified S-S-PEGylated histamine-loaded diethylenetriamine QDs. The prepared completely decorated QDs exhibited high mono-dispersibility, high stability, high drug loading efficiency, and biocompatibility. Most importantly, the formed multifunctional HA-modified nanocarriers exhibited CD44 receptor-mediated endocytosis, which facilitated targeted bioimaging and other therapeutic benefits of the nanocarrier (Table 7) (Fu et al., 2019).

**TABLE 7:** Illustration of quantum dot-targeted CD44 cells.

QDs	Therapeutic agents	Types of study (*in vitro*/*in vivo*)	Cancer types	Cell lines	Animal model	References
Nitrogen CQDs	—	Both	Breast cancer	ARPE-19 and CHO	NSG mice	Karakoçak et al. (2021)
CQDs	—	*In vitro*	Human gland	HeLa and MCF-7	—	Zhang et al. (2016)
HA-cysteamine polymer-wrapped QDs	—	*In vitro*	Breast cancer	MDA-MB-231, and MCF-7	—	Wang et al. (2014)
S-S- PEG-diethylenetriamine QDs	Histamine	*In vitro*	Breast	MCF-7 and A549 cells	—	Fu et al. (2019)

### CD44-Engineered Nanogel for Cancer Therapy

**FIGURE 7:**
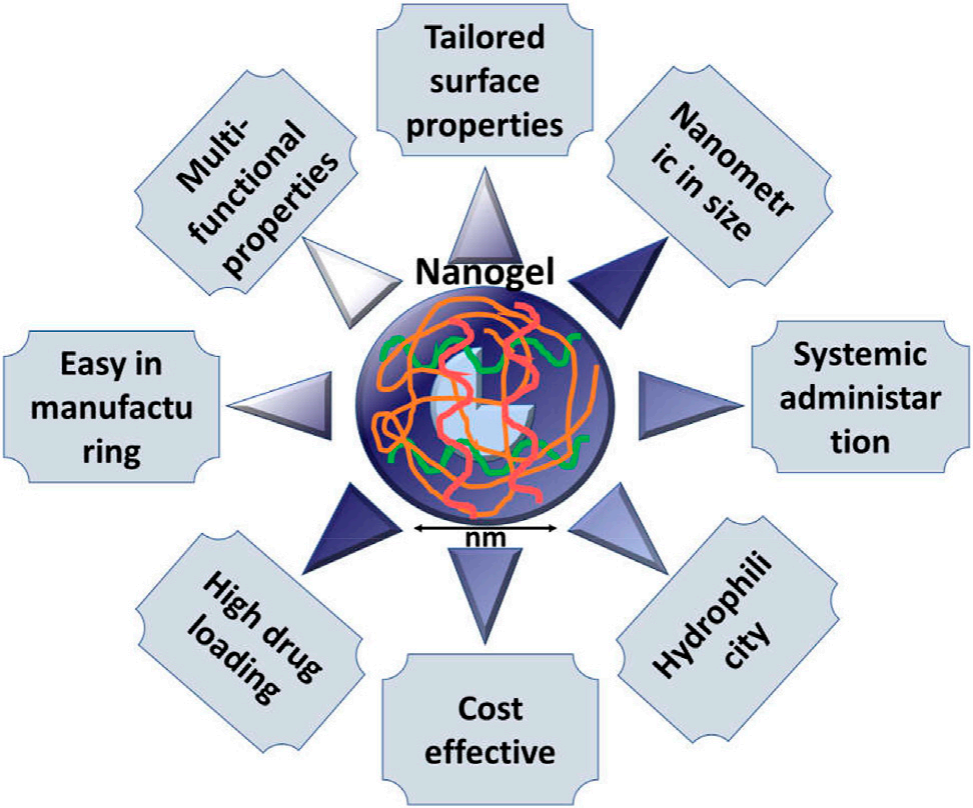
Schematic illustration of the different characteristics of nanogels.

Nanogels (NGs) are nanocarriers prepared by combining a hydrogel and a cross-linked hydrophilic polymer. NGs have attracted significant attention as promising drug-carrying agents for targeting cancer cells because they can exert unique properties such as greater functionality, easy formation, and improved targetability compared to traditional drug delivery systems. NGs are also known for some other features like high drug loading, better release capability, and easy tailoring (Cao Z et al., 2016; Nita et al., 2016; Qiao et al., 2016) (Figure 7). Wei and co-worker have designed and developed HA-based NG drug conjugates for the targeted delivery of etoposide (ETO), salinomycin (SAL), and curcumin (CUR). Here, the cholesteryl-hyaluronic acid (CHA) and non-HA nanogel was conjugated with ETO, SAL, and CUR by binding covalently through the degradable ester bond. Prepared CHA-drug conjugates exhibited particle sizes of 32.17 ± 5.66, 36.48 ± 6.23, and 29.15 ± 5.37 nm, respectively for CHA-ETO, CHA-SAL, and CHA-CUR. All preparations passively accumulated in the tumor cells *via* an enhanced permeability retention effect. *In vitro* cytotoxicity of CHA-drug conjugates was investigated in MDA-MB-231/F and MIA PaCa-2 cells, the IC50 value for CHA-ETO, CHA-SAL, and CHA-CUR was found to be 3.0, 0.9, and 9.0, respectively, which was comparatively better compared to free drugs. Notably, HA-modified drug nanogels exhibited about 2-7-fold greater cell cytotoxicity against MDA-MB-231/F and MIA PaCa-2 cells than that of free drugs and unmodified HA-drug conjugates. This difference in cytotoxicity results was attributed to the efficient cellular internalization of the HA-modified nanogel through CD44-mediated endocytosis. Also, after conjugation with cholesterol moieties, tumor accumulation of formulations was improved. The HA-modified drug nanogel was efficiently up taken into the multicellular spheroids and exhibited significantly higher anticancer activity in the modeling tumor environment as compared to free drugs and only HA drug conjugates, which means cholesterol played an important role into the activity of CHA-drug conjugates. Finally, it was concluded that the HA-modified drug nanogels prominently enhanced drug BA, tumor targeting, and therapeutic efficacy against resistant cancer cells (Wei et al., 2013). In similar study, Ma et al. used HA-based self-targeted nanogels for intracellular delivery of DOX and cisplatin to avoid MDR breast cancer. In this study, HA-based nanogels (HANGs) were coloaded with two anticancer molecules namely DOX and cisplatin. The formed nanogels exhibited intertumoral accumulation of drugs, synchronized pharmacokinetics, and prolonged blood distribution time. HA-modified nanogels loaded with both drugs exhibited efficient drug delivery into the multi-drug-resistant MCF-7/ADR breast cancer cells and exerted its anticancer activity intracellularly. By thoroughly understanding the mechanisms behind the internalization of HA-modified NGs into the MDR MCF-7/ADR tumor cells, researchers found that MCF-7/ADR and MCF-7 tumor cells overexpressed CD44 receptors, so that drug-loaded HA-modified NGs were efficiently internalized by tumor cells because of strong affinity of HA towards CD44 receptors which facilitated selective internalization of their cargo into CD44 + tumor cells. The nanogel avoided drug resistance *via* the following pathways, 1) the intracellular environment of tumor cells enriched with NGs due to specific internalization *via* CD44-mediated endocytosis leads to reduced P-gp efflux of NGs, 2) combination of DOX and cisplatin tackled clinical MDR, and 3) drugs were released at tumor pH because of disintegration of the compacted structure at acidic pH. This mechanism was confirmed by using the immunofluorescence staining and western blot technique. Finally, the researcher concluded that the HANGs had CD44 tumor targeting, reversing of MDR, and pH-responsive release effect for both drugs intracellularly (Ma et al., 2021). Seok et al. used a biodegradable, renewal, and cost-effective cross polymer called “Zein” for the preparation of a HA-modified nanogel for targeted delivery of anticancer drug curcumin (CRC). Here, the HA-coated Zein nanogel was prepared by the modified precipitation method. The formed HA-coated Zein NGs showed drug loading efficiency of 9.41% and drug encapsulation efficiency of 94.15%. *In vitro* selective cellular uptake was evaluated by a HA competitive assay, results of this assay revealed that the HA-coated Zein NGs were up taken *via* HA receptor-mediated endocytosis. *In vitro* anticancer efficacy of HA-coated Zein-CRC NGs was comparatively higher against CT26 cells than that of NIH3T3 cells. The IC50 value was 37 μg/ml and 94 μg/ml respectively, against CT26 and NIH3T3 cells. This anticancer efficacy difference of HA-coated Zein-CRC NGs between the CT26 and NIH3T3 cells was attributed to glutathione S-transferases (GST) level in the CT26 cells and also due to the level of CD44 receptors in the cells. *In vitro* stability and biocompatibility studies suggested that the HA-coated Zein-CRC NGs have sufficient stability in serum and compatibility of blood components. *In vivo* biodistribution of the conjugated HA-coated Zein-CRC NGs was investigated in a CT26 tumor model *via* conjugations with IR-780 dye. Higher tumor accumulation of HA-coated Zein-CRC NGs than Zein-CRC NGs led to improved therapeutic efficiency of CRC. These results were reconfirmed by histopathological analysis, which exhibited a higher apoptotic area in the HA-coated Zein-CRC NG-treated group than the untreated or free drug-treated groups. Finally, it was concluded that the CD44-targeted delivery of CRC by HA-coated Zein-CRC NGs could be a potential approach for anticancer therapy (Seok et al., 2018). Ma and co-worker designed and developed multifunctional HA-based NGs (HNPs). Here, all-trans retinoid acid (ATRA)/aggregation-induced emission luminogen (AlEgen) fluorophores were conjugated with HA *via* the disulphide bond. The developed NG was used for targeted delivery of DOX. The formed HA-modified NGs showed better drug loading efficiency with a sufficient stability profile at normal biological environments, DOX was rapidly released from the formulation in tumor microenvironments. *In vitro* cellular localization results revealed that the DOX was successfully transported into the cytoplasm of tumor cells through HNPs. This selective tumor accumulation of DOX-loaded HNPs was mainly attributed to targeted specific binding of HA to the CD44 receptor or LYCE-1 receptor. Higher intracellular accumulation of DOX-loaded HNPs caused significantly higher tumor cell suppression than the control and free drug. An *in vitro* real-time imaging biodistribution and uptake study of DOX-loaded HNPs exhibited CD44-mediated tumor accumulation and uptake in the tumor cells (Ma et al., 2018). In all the above studies, HA-decorated nanogels exhibited outstanding results which was due to binding of HA specifically to the CD44 receptor which promoted cellular internalization of nanocarriers, leading to release of their cargo into the tumor microenvironment because generally at low pH bonding between HA and its cargo could be broken. Importantly CD44-mediated internalization is the best way to bypass the Pgp reflux system and reverse MDR. Illustration of nanogel targeting CD44 cells is highlighted in Table 8.

**TABLE 8:** Outline of CD44-targeted therapy using nanogels.

Material	Therapeutic agents	Types of study (*in vitro*/*in vivo*)	Cancer types	Cell lines	Animal model	References
Cholesteryl chloroformate and HA	ETO, SAL, and CUR	*In vitro*	Breast	MDA-MB-231 and MIA PaCa-2	—	Wei et al. (2013)
FA	DOX and cisplatin	Both	Breast	MCF-7/ADR	BALB/c mice	Ma et al. (2021)
Zein	Curcumin	Both	Colon	CT26 and NIH3T3 normal cells	Mice	Seok et al. (2018)
HA-ATRA	Dox	*In vitro*	Hepatic	HepG-2 and 293 T cells	—	Ma et al. (2018)

## siRNA-Based CD44-Mediated Nanocarrier for Treatment of Various Cancers

**FIGURE 8:**
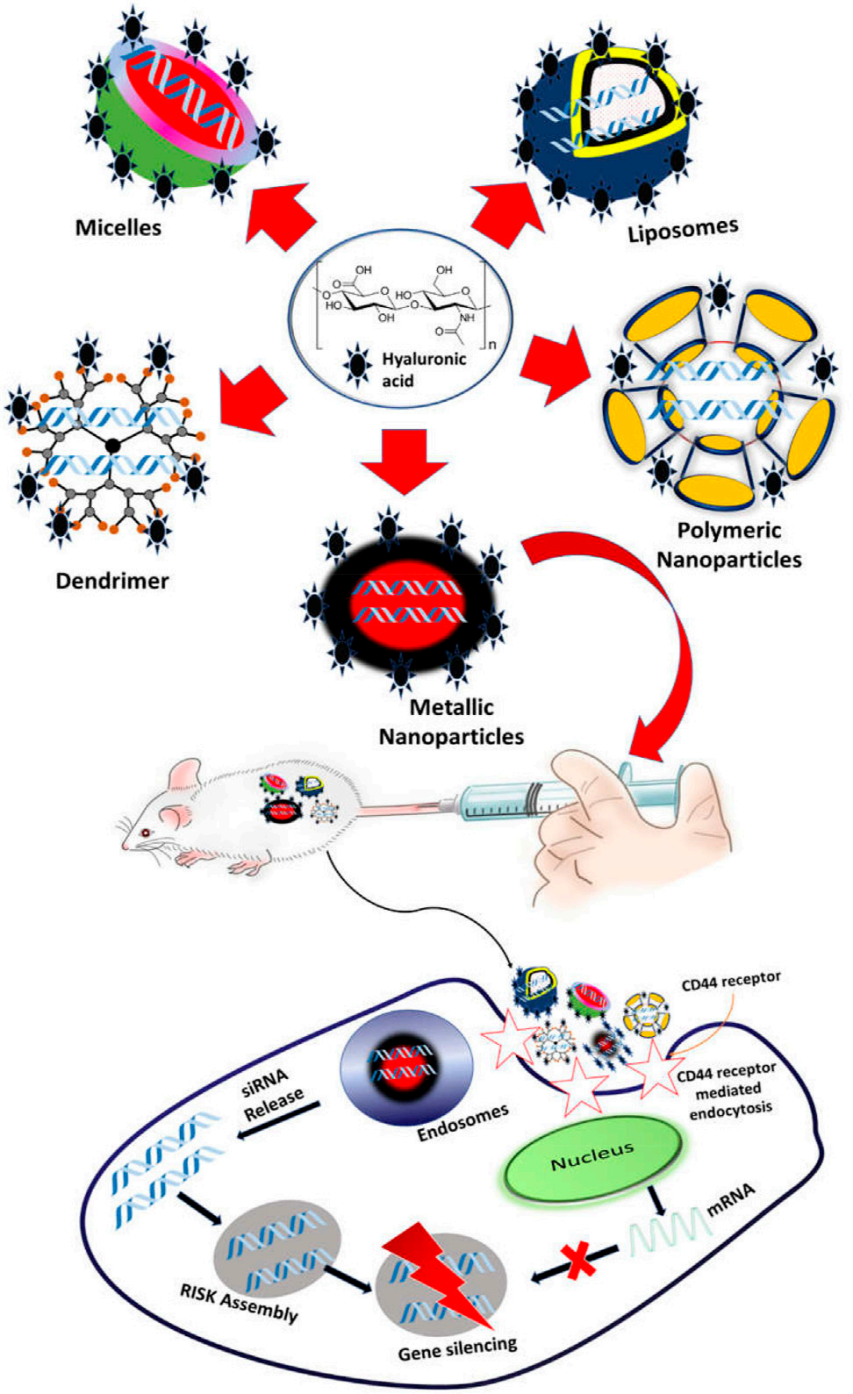
CD44-mediated siRNA delivery.

In the current scenario, due to potent, effective, and specific gene silencing effects, siRNAs have gained great recognition as therapeutic molecules for the treatment of many dangerous diseases like cancers, neurological diseases, etc. (Elbashir et al., 2001; Kim et al., 2008). However, translation of siRNA in clinical use poses a major challenge because naked siRNA possesses several intrinsic problems like poor cellular membrane penetration, inadequate stability in biological fluids, and most importantly a lack of targetability. To overcome this problem, numerous nanocarriers have been designed depending on the viral and non-viral vectors (Oishi et al., 2005). Use of a viral vector for the siRNA delivery is limited due to potential immune response of viral vectors, thus, researchers now focus more on non-viral vectors namely cationic liposomes, several polymers, and dendrimers, that could easily bind with the siRNA and deliver it to the targeted site (Figure 8) (Chen et al., 2009; Park et al., 2006). Moreover, by realizing the great potential of siRNA, it could be complexed with different nanocarriers that could impart the following properties, such as high targetability, high intracellular uptake, and site-specific release of siRNA (mostly into cytosol). In the following section, we discuss a few studies in which siRNA was delivered by a CD44-engineered nanocarrier, mostly by incorporating HA as a targeting ligand (Table 9) (Matsumoto et al., 2009; Kesharwani et al., 2015b). Yoon et al. has designed and developed a biodegradable, biocompatible HA cross-linked poly (dimethyl amino ethyl methacrylate) HPD conjugate which can easily form a complex with siRNA *via* the formation of the cross-linked disulfide bond. Gel electrophoresis results revealed among uncross-linked siRNA-HPD and cross-linked siRNA-HPD, crosslinked HPD efficiently formed a stable complex with the siRNA. An *in vitro* cell cytotoxicity study evaluated the conjugates in B16F10 and normal cells (NIH3T3 and CV-1 cell) and it was found that cross-linked siRNA-HPD caused a significance decrease in B16F10 cell viability at the maximum concentration >10 μg/ml while no observable viability changes were observed in CV-1 and NIH3T3 cells. Obtained results showed that the cytotoxicity of the complexes was regulated by expression of the CD44 receptor on cell lines. *In vitro* cellular uptake and gene silencing exhibited the best fluorescent signal for both cross-linked siRNA-HPD and uncross-linked siRNA-HPD in the B16F10 cells but an insignificant fluorescence signal was obtained in the NIH3T3 cells, similar types of results were also observed in an *in vivo* study. The *in vivo* fluorescence signal for both cross-linked siRNA-HPD and uncross-linked siRNA-HPD was reported in the initial 3 h due to the complex in the circulation, but after 3 h, the intensity of the fluorescence signal in the tumor site increased gradually, this may be due to HA-mediated selective uptake *via* the CD44 receptor at the tumor site. Importantly cross-linked siRNA-HPD always exerted a stronger signal than uncross-linked siRNA-HPD. A real-time NIRF technique revealed better *in vivo* gene silencing efficacy of both complexes in RFP-B16F10 tumor-bearing mice. The cross-linked siRNA-HPD complex showed around 6.6-fold less fluorescence intensity than that of control in the excised tumor. Finally, it was concluded that the cross-linked siRNA-HPD complex exhibited the best therapeutic results mainly *via* HA receptor-mediated endocytosis (Yoon et al., 2013). Shah et al*.* has designed a CD44-targeted nanocarrier for the delivery of siRNA and anticancer drug PTX for the purpose of CD44 cell suppression and cancer cell death initiation in ovarian cancer. The anticancer drug PTX was first linked to the PPI dendrimer *via* succinic acid as a spacer, additionally, luteinizing hormone–releasing hormone (LHRH) peptide as the targeting ligand was conjugated to the PEG polymer which surrounds the DTBP-caged dendrimer. Then, the PPI dendrimer was complexed with siRNA (PPI-siRNA). The LHRH–PPI–siRNA and LHRH–PPI–paclitaxel complexes showed decreased viability in ascitic cells around 10-fold that of control cells, > 5-fold that of the free PTX and >2 fold when compared to the non-targeted PPI–paclitaxel–siRNA complex. These results suggested the important role of LHRH peptide in mediating cellular cytotoxicity. Cellular uptake and distribution results showed significant higher fluorescence for PPI siRNA conjugated in cellular cytoplasm while very low fluorescence intensity was observed for naked siRNA in the tumor cell, which revealed that conjugated siRNA can easily penetrate and distribute intracellularly into the tumor cell, but naked siRNA cannot. In support of *in vitro* results, *in vivo* investigation of different formulations was carried out in nude mouse xenografts. The PPI-siRNA complex with HA suppressed the CD44 protein of the tumor cell while the other formulation did not show a significant change in expression of CD44 protein. Moreover, the PPI-siRNA conjugate exhibited extensive accumulation of dendrimer in the tumor cells, whereas the other formulation showed distribution in other tissues like liver, kidney, lungs, spleen, and heart. All the above results were attributed to the specific binding of LHRH peptide to the overexpressed CD44 receptor on tumor cells (Shah et al., 2013). Herrera et al. used ternary and quaternary polyplexes made up of siRNA-bPEI, modified with three different glycosaminoglycan (GAGs) polysaccharides, like HA, CS, and HA, alone or along with HSA for silencing of human mesenchymal stem cells (hMSCs) expressing green fluorescent protein. MSC or mesenchymal stem cells or multipotent stromal cells are employed in tissue repair sites and are also involved in immunomodulation, anti-inflammatory processes, and paracrine signaling. These cells are exceptional targets for genetic engineering. As reported in several studies, the N/P ratio plays an important role in stability and therapeutic efficiency of polyplexes, so here a balance between the polymer and siRNA was achieved at N/P 40 and above this. At an N/P ratio between 40–50, significant gene silencing of polyplex and stability in the cell cytosol condition was effectively obtained. As reported, there are several markers expressed on the hMSC surface and CD44 receptor. Thus, caveolae-mediated uptake of the whole polyplex is most probably driven by HA interactions with specifically overexpressed CD44 receptors on hMSC. Most importantly, increased cellular caveolae-mediated uptake of the HA-modified polyplex showed more than 97% GFP gene expression on hMSC cells (Herrera and Shastri, 2019). In another study, Xiong et al. designed and developed a codelivery system for anticancer drug DOX and a gene decorated with HA against hepatocellular carcinoma. The targeted nanoparticle showed enhanced uptake compared to the non-targeted nanoparticle. Similar to *in vitro* anticancer results, HA-modified NPs significantly showed higher accumulation of HA-modified NPs at the tumor site with maximum fluorescent intensity 24 h after injection. According to many previous studies, it was concluded that HA-modified NPs showed its better therapeutic effect due to selective binding of HA with CD44 receptor which was overexpressed on HCC cells (Xiong et al., 2017).

**TABLE 9:** Examples of siRNA-based CD44-mediated nanocarriers.

Material	Therapeutic agents	Types of study (*in vitro*/*in vivo*)	Cancer types	Cell lines	Animal model	References
HPD	siRNA	Both	Melanoma	B16F10 and NIH3T3	RFP-B16F10 tumor-bearing mice	Yoon et al. (2013)
LHRH–PPI	siRNA	Both	Ovarian	Ascitic cells	Female athymic nu/nu mice	Shah et al. (2013)
PEI with GAG and HSA	siRNA	*In vitro*	—	hMSCs	—	Herrera and Shastri, (2019)
Β-cyclodextrin- PLL	DOX and oligo RNA	Both	Hepatic cancer	MHCC-97H and HepG2	HCC xenograft mouse	Xiong et al. (2017)

## Chondroitin Sulfate and Chondroitin Sulfate-Based Nanocarrier

Among the types of glycosaminoglycans, CS is found in the extracellular matrix and plays a crucial role in cellular functions by modifying them. The structure of CS depends on the position of sulfation which is distinctively divided into four structures such as 1) chondroitin-4,6-sulfate, 2) chondroitin2,6-sulfate, 3) chondroitin-6-sulfate, and 4) chondroitin-4-sulfate. As a naturally occurring mucopolysaccharide, CS is extensively found in skin, cartilage, and bone. CS has high specificity towards CD44 receptors present on cancer cells. Due to such an effect, CS-modified nanocarriers have been formulated and have shown extraordinary benefit towards cancer therapy. A very recent study reported a docetaxel-loaded Zein nanoparticle modified with CS for effective treatment against prostate cancer. It is worth noting that the targeted preparation displayed improved pharmacokinetics with approximately a 9-fold half-life than free docetaxel. Moreover, a higher accumulation was observed in PC-3 tumor cell-bearing mice that indicated enhanced accumulation at the tumor site. Hence, a CS-based nanoparticle imparts the hope of effective therapy (Lee et al., n.d.). Another study reported a temozolomide-loaded albumin nanoparticle fabricated with CS for brain-targeted delivery. The preparation was characterized for surface charge, particle size, DSC, XRD, FTIR, and TEM, and revealed suitable results. Moreover, the biodistribution results exhibited a higher accumulation of drugs in the brain by the targeted preparation as compared with the pure drug that significantly confirmed the brain targeting ability of nanoparticles (Kudarha and Sawant, 2021). Oommen et al. confirmed the anti-cancer effect against breast and colon cancer cells by using CS and HA nanoparticles loaded with doxorubicin (Oommen et al., 2016). Luo et al. explored the targeting effect of a CS-modified DOX and retinoic acid-loaded lipid nanoparticle against liver cancer. The resulting preparation was found to be accumulated in Golgi bodies and also inhibited the ECM production proving its targeting potential against the most dangerous cancer (Luo et al., 2020). Hence, to obtain a profound therapeutic effect with low toxicity and off-site effect, a targeting therapy based on CS and HA is highly effective.

## Conclusion and Future Perspective

Active targeting holds promise regarding target identification, efficient tumor cell death, and reduced off-target effect and toxicities of chemotherapeutic agents. Nowadays, interest has been increasing towards targeted delivery of chemotherapeutics to design effective and efficient formulations. The CD44 receptor is the most expressed receptor on several tumor cells and it has a great affinity towards two polymers HA and CS which are natural targets for formulations. A nanocarrier modified by HA/CS coating or used as a targeting ligand/moiety could be the backbone of a nanocarrier against CD44. In the current review, many nanocarriers such as dendrimers, nanoparticles, micelles, nanoemulsions, CNTs, quantum dots, nanogels, and other polymeric carriers coated with HA/CS can effectively/efficiently binding with the CD44 receptor facilitating cellular internalization of nanocarriers loaded with cargo into the tumor cells. In some reports, CD44-mediated endocytosis could enable the bypass of MDR or the Pgp system. In several cases, the CD44 receptor had a significant role in intracellular and safe delivery of several genes against the most dangerous and fatal cancers. The CD44 receptor can be targeted by various agents such as antibodies, single chain variable fragments, peptides, and macromolecules such as hyaluronic acid and chondroitin which show a tumor homing effect, sparing the normal cells. Thus, the toxicity of treatment could be reduced. However, the major limitation is the heterogeneity of the tumor cells that show different isoforms of CD44. Thus, a thorough study of proteomics is required to overcome such pitfalls. Thus, it could be established that the CD44 receptor has great potential as a therapeutic target against several cancers.
